# The effects of a 6-week intervention with *Limosilactobacillus reuteri* ATCC PTA 6475 alone and in combination with *L*. *reuteri* DSM 17938 on gut barrier function, immune markers, and symptoms in patients with IBS-D—An exploratory RCT

**DOI:** 10.1371/journal.pone.0312464

**Published:** 2024-11-01

**Authors:** Julia König, María Fernanda Roca Rubio, Richard A. Forsgård, Julia Rode, Jakob Axelsson, Gianfranco Grompone, Robert J. Brummer

**Affiliations:** 1 Nutrition-Gut-Brain Interactions Research Centre, Faculty of Health and Medicine, School of Medical Sciences, Örebro University, Örebro, Sweden; 2 BioGaia AB, Eslöv, Sweden; 3 BioGaia AB, Stockholm, Sweden; Fonterra Coop / Lebanese University, LEBANON

## Abstract

**Background:**

An increased intestinal permeability is a common feature in patients with diarrhoea-predominant irritable bowel syndrome (IBS-D). Probiotics have shown to improve IBS symptoms and might also affect intestinal barrier function.

**Aim:**

The aim of this study was to investigate the effects of a 6-week intervention with *Limosilactobacillus reuteri* ATCC PTA 6475 alone (single strain) or in combination with *Limosilactobacillus reuteri* DSM 17938 (dual strain) on gut barrier function, immune markers, and symptoms in IBS-D patients (ClinicalTrials.gov registration number: NCT03986476).

**Methods:**

65 IBS-D patients were randomised into three groups (placebo, single strain, dual strain). Small and large intestinal permeability were assessed using a multi-sugar urinary recovery test. Blood, saliva, faecal samples, and several symptom scales were collected before, and after three and six weeks of intervention.

**Results:**

Small and large intestinal permeability as well as other markers of gut barrier function were not significantly affected by the probiotic interventions. Serum IL-6 levels showed a tendency to be reduced in the single strain group (descriptive p = 0.052). In addition, high-sensitivity C-reactive protein was significantly reduced in the dual strain group (p = 0.041). The participants in both treatment groups reported less gastrointestinal symptoms after three weeks, but this reached significance only in the dual strain group (total score: p = 0.032, pain subscore: p = 0.028). After six weeks, none of the assessed symptoms were significantly different from the placebo.

**Conclusion:**

The probiotic compounds investigated in this study did not seem to affect IBS-D patients’ gut barrier function, but showed potential anti-inflammatory and symptom-improving properties, which need to be confirmed in larger study cohorts.

## Introduction

Irritable bowel syndrome (IBS) is a common disorder of gut-brain interaction [1]. Even though it is not life-threatening or associated with higher mortality, IBS profoundly affects the patients’ quality of life and is associated with substantial economic costs due to the need for medical consultation and work absenteeism [2]. The aetiology and pathophysiology of IBS are complex and not well understood, but it is accepted that a dysregulation of the microbe-gut-brain axis plays an important role [1]. Associated aberrations include visceral hypersensitivity, abnormal gut motility, gut microbiota changes, and autonomic nervous system dysfunction [3, 4]. In addition, mild immune activation has been found both locally in the gut and systemically in a subgroup of patients [5–7]. Specifically among patients with diarrhoea-predominant IBS (IBS-D), a compromised intestinal barrier function appears to be common [8]. The intestinal barrier is an important component in maintaining gut homeostasis and human health. It prevents translocation of harmful substances and microbial products from the external environment while allowing a peaceful coexistence with intestinal symbionts without causing chronic inflammation [9]. Disruption of this barrier can result in increased intestinal permeability, which in turn triggers local immune activation and facilitates translocation of harmful products to the bloodstream. Intestinal barrier dysfunction has been related to several diseases, such as food allergies, coeliac disease, obesity, and inflammatory bowel disease (IBD) [9]. Also, psychological stress, which is often increased in IBS patients, has been suggested to enhance intestinal permeability via the hypothalamic-pituitary-adrenal (HPA) axis and mast cell activation [10].

Studies using sugar probes to assess intestinal permeability have shown that between 37% and 62% of IBS-D patients have an increased small intestinal permeability [8]. Additionally, *ex vivo* experiments have revealed that colon biopsies of IBS patients display an increased permeability and release soluble factors that can reduce barrier function in other cells [11, 12].

Several meta-analyses have shown that the intake of specific beneficial bacteria, so-called probiotics, can improve gastrointestinal symptoms of IBS patients [13, 14]. In addition, there is evidence that administration of probiotics can positively affect stress responses [15–17]. The mechanisms behind the beneficial effect of probiotics are still mostly unknown, yet one suggested mechanism could be a strengthening of the barrier function. A study showed that probiotics administered by a feeding tube strengthened the intestinal barrier both in healthy volunteers as well as in IBS patients [18]. The strains *Limosilactobacillus reuteri* ATCC PTA 6475 and *Limosilactobacillus reuteri* DSM 17938 used in the presented study have shown beneficial effect on the intestinal barrier in several cell and animal studies [19–21]. Furthermore, both strains have shown complementary immune-modulatory and anti-inflammatory properties *in vivo*, which can potentially lead to strengthening of the intestinal barrier and might have beneficial effects in IBS [22–29]. Therefore, a combination of these two strains has been investigated in different conditions of gastrointestinal complaints and inflammatory states. In a recent randomised clinical trial, the combination of these strains combined with standard care administered for 14 weeks improved symptoms and faecal calprotectin levels in patients with moderate to severe IBS compared to the placebo [30]. When used as an adjunctive therapy to *Helicobacter pylori* eradication, the strain combination significantly reduced gastrointestinal symptoms, such as abdominal pain/distension, bloating, epigastic pain, and diarrhoea [31–33].

The primary aim of this exploratory study was to investigate the effect of a 6-week intervention with *L*. *reuteri* ATCC PTA 6475 alone or in combination with *L*. *reuteri* DSM 17938 on the small intestinal permeability of IBS-D patients. In addition, the effect of these probiotics on different markers of barrier function, immune response, and stress as well as on IBS symptoms was investigated. Our results showed that, while markers of intestinal permeability and barrier function were not significantly affected by the investigated probiotic strains, they might have an effect on markers of immune response and gastrointestinal symptoms in this IBS-D cohort.

## Methods

### Ethical statement

The study was performed according to the Helsinki declaration and its revisions. It was approved by the Regional Ethics Review Board of Uppsala, Sweden (registration number 2018/450, amendment by the Swedish Ethical Review Authority 2022-01726-02) and reported according to the CONSORT 2010 checklist (S1 Table). The study was performed at Örebro University in Örebro, Sweden, from March 2019 until June 2021 and registered at ClinicalTrials.gov (NCT03986476) on June 14, 2019. The delay was due to an oversight in the administrative processes by the responsible researcher. Individual written consent was obtained from all individual participants included in the study.

### Sample size calculation

The sample size was based on the ability to find a significant lactulose/rhamnose ratio difference between one of the probiotic study arms and the placebo arm. A change of 15% in lactulose/rhamnose ratio was considered as clinically relevant and should be detected as significant, which according to a study by Zuhl et al. (2014) amounts to 0.009 points in ratio difference [34]. Based on this and a power of 80%, an expected standard deviation of 0.01, a significance (alpha) level of 5%, two-sided testing, and an estimated drop-out rate of 15%, a sample size of 75 participants (n = 25 per group) was calculated.

### Participants

Patients with IBS-D were recruited through advertisement placed at Örebro University, in the local newspaper and social media. A webpage was set up where interested participants could sign up (www.oru.se/ibs-d). Interested participants were invited for an information visit held at Örebro University. After providing written informed consent, subjects completed screening procedures to evaluate their eligibility for the study. Inclusion criteria were an age between 18 and 65 years, mild to severe IBS symptoms according to the IBS severity scoring system (IBS-SSS score of ≥75), fulfilled Rome IV diagnostic criteria for IBS [35] as well as IBS-D (more than 25% loose stools and less than 25% hard stools, assessed by questionnaire). Exclusion criteria included, amongst others, gastrointestinal or autoimmune diseases, previous abdominal surgery (except appendectomy and cholecystectomy), recent or current use of medications that potentially affect the intestinal permeability or gut microbiota such as antidepressants, non-steroidal anti-inflammatory drugs (NSAIDs), and probiotics (a complete list of exclusion criteria can be found in S2 Table).

### Study outcomes

The primary endpoint was defined as the difference in small intestinal permeability, measured as the urinary lactulose/rhamnose secretion ratio, after a 6-week intervention with the probiotic products compared to the placebo product, in IBS-D patients.

Secondary endpoints were defined as the differences between intervention with the probiotic products compared to the placebo in IBS-D patients regarding small intestinal permeability, measured as the urinary lactulose/rhamnose secretion ratio, after 3-week intervention; whole gut permeability measured as urinary sucralose/erythritol excretion ratio (0–24 hours) after 3 and 6 weeks; colonic permeability measured as urinary sucralose/erythritol ratio (5–24 hours) after 3 and 6 weeks; gastroduodenal permeability measured as urinary sucrose excretion (0–5 hours) after 3 and 6 weeks; and regarding various potential biomarkers of general intestinal barrier function and inflammation in blood and faecal samples after 3 and 6 weeks. After completion of the trial, we decided not to include whole gut permeability as it was better represented by separately reporting small and large gut permeability only. Gastroduodenal permeability was excluded due to technical problems with the analyses.

### Study design

In this double-blinded, randomised, placebo-controlled study, participants were scheduled for five visits, of which the first visit was a screening visit. After a run-in period including two baseline visits separated by three weeks, participants were randomised to a six-week intervention with one of the probiotic products or the placebo, with follow-up visits three weeks and six weeks after start of the intervention. The visits were performed at similar hours of the day to avoid diurnal hormonal changes. At all visits, participants provided urinary, blood, faecal, and saliva samples and completed symptom scales. During the entire period of the study, participants completed a stool diary based on the Bristol stool chart and were asked to avoid changes in their diet and lifestyle, such as exercise and sleeping habits. Participants were carefully instructed to refrain from taking any NSAIDs, antibiotics, anticholinergic drugs, oral mast cell stabilising drugs, and pre- and probiotics during the entire study period. Study participants were also asked to avoid strenuous exercise, intake of the artificial sugars used in the multi-sugar test, alcohol, nicotine, and spicy foods two days prior to as well as during each multi-sugar test. Participants were also asked to refrain from caffeine during the urine collection. In addition, participants were asked to consume the same meal the evening before each multi-sugar test.

### Study products

The study products consisted of capsules containing either *L*. *reuteri* ATCC PTA 6475 alone (‘single strain’) or in combination with *L*. *reuteri* DSM 17938 (‘dual strain’). The placebo product consisted of identical ingredients except for the absence of probiotics. All study products were indistinguishable in packaging, appearance, and taste. Participants were asked to consume one capsule twice a day, one with breakfast and one with dinner, giving a daily dose of a minimum of 1 x 10^9^ CFU of the single strain product and 2 x 10^9^ CFU of the dual strain product (1 x 10^9^ CFU of each strain). The CFU counts of the active products were monitored throughout the entire study period and were at all times within specifications.

### Compliance

Compliance was assessed using a diary as well as based on the returned study product. In case of discrepancy between these measures, the lower compliance was used. Participants that consumed less than 80% were excluded from the analysis.

### Randomisation and masking

Study participants were randomly assigned to a six-week intake of either the single strain, the dual strain, or placebo in a ratio of 1:1:1 (stratified by sex) by an independent researcher using a computerised randomisation list applying block randomisation with a random block size of six and four. All participants and investigators remained blinded until the statistical analyses of the outcomes and the symptom scale data were completed. Containers with the study products were labelled with sequential allocation numbers to conceal their content and to ensure blinding of participants and investigators.

### *In vivo* intestinal permeability test

At all baseline and follow-up visits, the intestinal permeability was measured using a standardised multi-sugar test, which is a non-invasive, sensitive technique suited to detect small changes in small and large intestinal permeability [36, 37]. For this procedure, the participants consumed, at home, after an overnight fast, 150 ml of tap water containing 1 g sucrose (Nordic Sugar, Sweden), 1 g lactulose (Solactis, France), 1 g sucralose (HYET Sweet, Netherlands), 1 g erythritol (HYET Sweet, Netherlands) and 0.5 g rhamnose (Sigma-Aldrich MO, USA). This was followed by total urinary collection for 24 hours. Urine was collected in two different urine containers (Sarstedt, Sweden), one for 0–5 hours collection, and one for the following 5–24 hours collection, and stored in cooling bags. During the first five hours of urine collection, the participants abstained from the intake of food, and were urged to drink minimum 1.5 litres of water. After the 5-hour collection period, subjects aliquoted 4 ml of the content into two V-monovette urinary tubes (Sarstedt, Sweden), and stored them in their home freezer. Participants then continued to collect their urine for the remaining 19 hours in the second urine container. Subsequently, subjects aliquoted 4 ml of the content of the second fraction into two V-monovette urinary tubes (Sarstedt, Sweden) and stored them in their home freezer. The study participants returned the frozen urine samples to the university staff at the next study visit. The urine samples were stored at -80°C until further analysis. A total of 5 μl of urine sample was used to detect the urinary excretion ratios of the sugars. The analysis was performed using UPLC-MS/MS as previously described [38, 39]. The ratio of recovered lactulose to rhamnose (L/R) in 0–5 hour urine represented small intestinal permeability whereas colonic permeability was assessed by the ratio of sucralose to erythritol (S/E) in 5–24 hour urine [36–40].

### Plasma markers of gut barrier function and inflammatory status

Blood was collected at all baseline and follow-up visits after an overnight fast. After centrifugation, serum, Lithium heparin and EDTA plasma samples were aliquoted into cryogenic vials and frozen at -80°C until analysis. Plasma samples for the analysis of high- sensitivity C-reactive protein (hsCRP) were directly delivered to the Department of Laboratory Medicine at Örebro University Hospital for analysis according to clinical routines.

#### Intestinal fatty acid binding protein (I-FABP)

I-FABP concentrations in plasma were analysed using an ELISA (HK406, HycultBiotech, Uden, The Netherlands) following the manufacturer’s instructions.

#### Lipopolysaccharide binding protein (LBP)

LBP plasma concentrations were analysed using an ELISA (HK315, HycultBiotech) following the manufacturer’s instructions.

#### High sensitivity C-reactive protein (hsCRP)

hsCRP concentrations were measured by a latex enhanced immunoturbidimetric assay (ADVIA^®^ Chemistry CardioPhase^™^ High Sensitivity C‑Reactive Protein, Siemens Healthcare Diagnostics Inc) according to clinical routines by the Department of Laboratory Medicine at Örebro University Hospital. Normal values in adults were specified as <3.0 mg/L.

#### Cytokines

Serum concentrations of interleukin (IL)-4, IL-5, IL-6, IL-8, IL-10, interferon-gamma (IFN-γ) and tumour necrosis factor-alpha (TNF-α) in serum were assessed through a V-Plex proinflammatory panel 1 (Meso Scale Diagnostics, Rockville, MD, USA) following manufacturer’s instructions. Data regarding IL-1ß and IL-12p70 were not shown as most values were below detection levels.

### Faecal calprotectin

Faecal samples were collected at home using provided faecal sample tubes before each visit. The samples were immediately placed into the home freezer by the subjects and returned frozen to the study unit using special cool transport containers (Sarstedt, Germany). At the study unit the faecal samples were stored at -80°C until further analysis. Before analysis, stool samples were thawed and transferred to faecal extraction devices (BÜHLMANN CALEX^®^ Cap, BÜHLMANN Laboratories AG (BL), Basel, Switzerland). Samples were analysed according to clinical routine by the Department of Laboratory Chemistry, Örebro University Hospital using a latex enhanced immunoturbidimetric assay (Calprotectin Turbilatex, Advia 1800, Siemens Healthcare AB, Erlangen, Germany). Normal values in adults were specified as <80 mg/kg.

### Salivary cortisol awakening response including cortisol at awakening

Saliva samples were collected at home as previously described before all visits [41]. In short, participants collected five samples within one hour after awakening in the morning on three consecutive days (two weekdays, one weekend day): Directly after awakening and at 15, 30, 45 and 60 minutes thereafter. Samples were stored in the home freezer until delivery to the study centre using cooling packs. The three samples from one respective time point were pooled before analysis [42]. Salivary cortisol levels were measured using a commercially available chemiluminescence immunoassay with high sensitivity (IBL, Hamburg, Germany) at DresdenLab Service GmbH (Dresden, Germany) as previously described [41]. The intra- and interassay coefficients for cortisol were both below 9%. The area under the curve with respect to increase (AUCi) was calculated as recommended by Stalder et al. 2016 [43]. In addition to the AUCi, the levels of the first cortisol sample directly after awakening were reported separately, as recommended by Stalder et al. 2016 [43].

### IBS symptom assessment

The IBS-Symptom Severity Scale (IBS-SSS), developed by Francis et al. [44], was used to evaluate the severity of the patients’ IBS symptoms at all study visits. This tool is a well validated system to assess severity of symptoms in IBS patients [45]. The scoring system includes five items and analyses stool frequency, stool consistency, abdominal pain, distension and interference with daily life in a ten-day period [44]. Each item is scored on a visual analogue scale from 0 to 100, the sum is the total score with a maximum achievable score of 500. A score between 75 and 175 corresponds to mild IBS symptoms, a score in the range of 176–300 describes moderate IBS symptoms, and scores above 300 correspond to severe IBS symptoms.

The Gastrointestinal Symptom Rating Scale (GSRS)-IBS is a validated IBS-specific symptom scale [46] and was completed at all study visits. It includes 13 items in five symptom clusters (abdominal pain, bloating, constipation, diarrhoea, and satiety) and uses a seven-point Likert scale ranging from 1 (no discomfort at all) to 7 (very severe discomfort) to measure symptoms during the past seven days.

### Hospital anxiety and depression scale (HADS)

The HADS was originally developed by Zigmond and Snaith for use in a medical setting, and its validity and reliability has been reported in several studies. It was completed at all study visits and consists of 14 items divided into two subscales for anxiety (seven items) and depression (seven items) [47]. Patients rate each item on a four-point scale (0–3). For each subscale, scores higher than 11 imply definite cases of anxiety or depression, respectively, a range of 8–10 a probable case, and ≤ 7 no case.

### Self-rated health status

The EQ-5D-5L is a measure of health status by the EuroQol group that provides descriptive measures of the five dimensions mobility, self-care, usual activities, pain/discomfort, anxiety/depression as well as the health status [48]. In this study, only the quantitative health status was used, for which participants rate their current health status on a visual analogue scale, with 100 being the best health status possible (EQ-VAS). It was completed before and 6 weeks after start of the intervention.

### Bristol stool chart diary

During the entire study period, participants were asked to complete a diary based on the Bristol stool chart, in which they reported each defaecation and its consistency. Stool frequency and consistency were calculated as the daily average per week. The average of the baseline data from three weeks was calculated and subtracted from all other values. If less than four days per week were available, the whole week was reported as missing values. Data reported on days of the multi-sugar test were excluded.

### Data analysis

Kruskal-Wallis test with Mann-Whitney U as post-hoc test was used for comparisons of baseline characteristics, as most of this data was not normally distributed (tested with Shapiro-Wilk test). Fisher’s exact test was used for categorial data. Intervention data were baseline-corrected, and the three weeks and the six weeks data of the single and the dual strain separately compared to placebo. The average of the data of the two baseline visits was used. To account for baseline differences in age and L/R, one-way analysis of covariance (ANCOVA) was performed with age and baseline L/R as covariates, group as the independent variable and log-transformed, baseline-corrected outcome data as dependent variables. Data was log-transformed to normalise the distribution of the data. If the data sets contained negative or zero values, a constant value was added to all values in the data set. Prior to conducting the ANCOVA, several assumptions were tested to ensure the validity of the analysis. Outliers were defined as z-scores > 3.0 and removed from the data set before analysis. The data set was tested for normal distribution of residuals using the Shapiro-Wilk test of normality, and for homogeneity of variances using the Levene’s test. Also homogeneity of regression slopes was tested. The data set passed all these tests (no significant findings after multiplicity assessment using FDR-correction by Benjamini-Hochberg). Homoscedasticity was confirmed by visual inspection of scatterplots of the standardized residuals against the predicted values.

Data was analysed using pairwise deletion, i.e., missing values were not replaced, and all available data was included in the analyses even if a participant had one or more missing values. Multiple imputation was performed for primary outcomes as well as secondary outcomes with p-values <0.06, and was conducted using the automatic method in SPSS, generating five imputed datasets. For continuous variables, a linear regression model was employed to estimate missing values. No interactions were included in the imputation models. The imputation process handled variables with up to 100% missing data, and a maximum of 100 parameters were included in the imputation model. Pooled values of all five imputations were reported.

After correction for multiplicity using the Benjamin-Hochberg FDR procedure, no results were significant. Due to the exploratory character of this study, descriptive p-values were reported. Post hoc power analyses were performed using the actual mean values and standard deviations obtained in the study, based on a power of 80%, and a significance (alpha) level of 5% with two-sided testing. All calculations were performed using SPSS Statistics Version 29.0.2.0 (IBM, Armonk, NY, USA), apart from the analysis of the stool diary data which was analysed by fitting a mixed model as implemented in GraphPad Prism 9.2 (GraphPad Software Incorporated, La Jolla, CA, USA). This mixed model was applied to account for the many time points and uses a compound symmetry covariance matrix, is fit using Restricted Maximum Likelihood (REML), and Geisser-Greenhouse correction was applied.

## Results

### Baseline characteristics and adverse events

**Fig 1:**
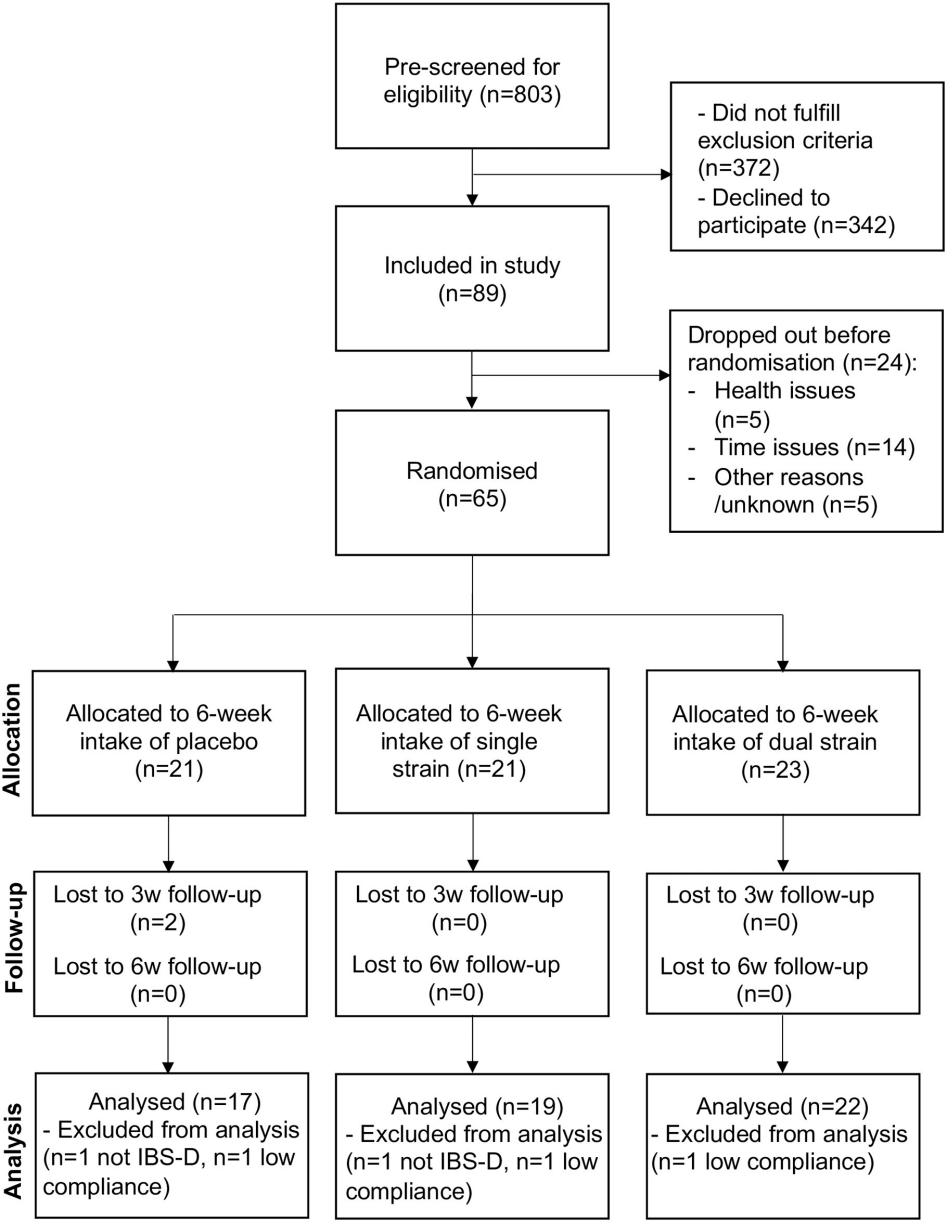
CONSORT flow diagram. IBS-D—Diarrhoea-predominant irritable bowel syndrome.

From a total of 803 interested study participants, 89 were included in the study, of which 65 were randomised into three groups (for Consort flow chart see Fig 1). Two participants randomised to the placebo group discontinued the study before the three weeks follow-up visit, one due to time issues and one for unknown reasons. One participant in the placebo group and one in the single strain group were excluded from the analysis as they did not qualify as IBS-D according to the diary data from the run-in period (inclusion based on interview). In addition, one participant in each group was excluded from the analysis due to low compliance. One participant in the dual strain group did not collect urine samples (for the assessment of the primary outcome) nor faecal samples but provided blood samples and completed symptoms scales. Available data from this participant was included in the analysis. An overview of protocol violations leading to exclusion of data is shown in S3 Table.

Baseline characteristics of the participants included in the analysis are shown in Table 1. About two thirds of the participants were women (38 out of 58). The median age of the participants randomised to the dual strain group (27 years) was significantly lower compared to the placebo group (44 years, p = 0.026) and the single strain group (44 years, p = 0.041). Also, baseline small intestinal permeability (L/R) was significantly lower in the dual strain group compared to the single strain group (p = 0.038), but not compared to the placebo group (p = 0.130). Hence, all data was analysed using one-way analysis of covariance (ANCOVA) with age and baseline L/R as a covariates. n = 1 baseline L/R value was missing in the data set, this missing value was imputed with the average of all L/R baseline values. Unadjusted results for those outcomes for which the covariates had a significant effect can be found in S4 Table.

**Table 1:** Baseline characteristics of study participants.

		Placebo (n = 17)	Single strain (n = 19)	Dual strain (n = 22)
**Females/males,** n (%)		12 (70.6)/5 (29.4)	12 (63.2)/7 (36.8)	14 (63.6)/8 (36.4)
**Age**, years [median (min-max)]		44 (19–63)	44 (20–63)	27 (19–58)^1^
**BMI**, kg/m^2^ [median (min-max)]		24.2 (17.4–35.0)	24.0 (20.0–37.2)	24.9 (17.6–33.4)
**Duration IBS**, % (0.5-1/1-5/>5 years)		0.0/5.6^2^/94.1^3^	21.1/21.1/57.9	0/36.4/63.6
**PI-IBS**, % (Possibly yes/possibly no)		5.9/94.1	21.1/79.0	4.6/95.5
**IBS-SSS scores**, [median (IQR)]		260 (196–279)	248 (203–298)	240 (174–308)
**GSRS total scores**, [median (IQR)]		3.5 (2.7–3.8)	3.2 (2.5–4.0)	3.4 (2.8–3.7)
**HADS anxiety scores**, [median (IQR)]		6.5 (2.5–9.3)	8.0 (3.5–9.5)	6.8 (4.5–9.8)
**HADS depression scores**, [median (IQR)]		2.5 (0.8–5.0)	4.5 (1.5–6.5)	4.0 (1.4–5.0)
**Health status**, (EQ-VAS), [median (IQR)]		68 (60–90)	73 (65–80)	73 (66–78)
**L/R**, [median (IQR)]		0.042 (0.030–0.049)	0.042 (0.033–0.065)	0.029 (0.025–0.046)^4^
**Increased L/R compared to healthy**^5^, n (%)		11 (65)	15 (79)	10 (48)
**S/E**, [median (IQR)]		0.028 (0.023–0.032)	0.030 (0.026–0.034)	0.030 (0.022–0.043)
**Faecal Calprotectin**, mg/kg, [median (IQR)]		37.8 (21.8–67.8)	26.0 (14.5–52.0)	34.0 (21.5–57.9)
**hsCRP**, mg/L, [median (IQR)]		0.9 (0.5–1.9)	1.2 (0.5–2.0)	2.8 (0.9–3.8)
**IL-6**, mg/L, [median (IQR)]		0.60 (0.41–0.78)	0.53 (0.34–0.69)	0.52 (0.36–0.73)
**Tobacco use**, n (yes/sporadically)		0/2	0/1	1/0
**Special diet**, n	**Total**	7	9	12
	Lactose free	1	2	5
	Vegan	1	1	1
	Vegetarian	3	2	3
	Avoiding certain foods	2	3	2
	Low FODMAP	0	1	1
**Comorbidities**, n	**Total**	5	7	13
	Allergies	3	1	6
	Dyspepsia	1	1	2
	Migraines	0	1	0
	Lactose intolerance	0	0	2
	Asthma	1	2	2
	Diverticulosis	0	1	0
	Hypertension	0	1	0
	Chronic back pain	0	0	1
**Concomitant IBS medication**, n	**Total**	4	1	4
	Anti-diarrhoeal	3	0	3
	Bulking	1	1	1
	Anti-foaming	0	0	0
**Concomitant other medication**, n	**Total**	3	5	5
	Analgesics	0	2	1
	Antiemetics	1	0	0
	Anti-fungal	1	0	0
	Asthma/allergy-related	1	2	2
	Contraceptives	0	1	0
	Iron substitution	0	0	1
	Proton pump inhibitors	0	0	1
	Sedatives	0	0	0

Kruskal-Wallis test with post-hoc Mann-Whitney U test was used for ordinal and Fisher’s exact test for categorical data.

^1^Significantly different compared to the placebo group (p = 0.026) and the single strain group (p = 0.041).

^2^Significantly reduced compared to the dual strain group (p = 0.027).

^3^Significantly increased compared to the single strain group (p = 0.020).

^4^Significantly reduced compared to the single strain group (p = 0.038).

^5^Small intestinal permeability, assessed as urinary recovery of L/R, was defined as increased when values were above the upper 95% confidence interval of the pooled values of two previously published studies with healthy controls using the same assessment method (>0.033) [38, 39].

Special diets and concomitant medications are presented as ‘n’ as multiple answers per participants were possible. IBS—irritable bowel syndrome. PI-IBS—post-infectious IBS. IBS-SSS—IBS-severity scoring system. IQR—interquartile range. GSRS—Gastrointestinal symptom scale. HADS—Hospital anxiety and depression score. EQ—EuroQol. VAS—Visual analogue scale. L/R—lactulose/rhamnose excretion ratio. S/E—sucralose/erythritol excretion ratio. hs-CRP—high sensitivity C-reactive protein. IL—interleukin. FODMAP—fermentable oligosaccharides, disaccharides, monosaccharides, and polyols.

IBS symptoms at baseline were of moderate severity in all three groups (placebo: median IBS-SSS score of 260 [interquartile range: 196, 279]; single strain: 248 [203, 298]; dual strain: 240 [174, 308]) and did not differ significantly between the groups (Table 1). In the placebo group, significantly less participants had IBS symptoms for 1–5 years compared to the dual strain group, while significantly more participants had experienced symptoms for more than 5 years compared to the single strain group. However, the majority of the participants in all groups had experienced symptoms for more than five years.

No serious adverse events were reported in any of the groups, and there were no significant differences in number of adverse events between the groups (see S5 Table).

### Missing values

Due to the outbreak of COVID-19, we were not able to collect all samples as planned since participants had to cancel study visits when showing possible symptoms. This affected especially the blood sampling. During the early phase of the pandemic, we decided to cancel many three-week follow-up blood sampling visits to minimise contact between participants and study staff. Participants were instructed to collect all other samples at home and store them in their home freezer at -20°C until attending the six-week follow-up visit. This decision affected especially the dual strain group (S6 Table). In addition, some data was missing due to other reasons unrelated to treatment or IBS status (e.g., not possible to draw blood, participant did not collect urine samples, severe protocol violations). At the six weeks visit, this affected mainly the single strain group, where blood values were missing for the following reasons: delayed sampling (n = 1), not possible to draw blood (n = 1), technical error (n = 1), suspected COVID-19 infection (n = 1), low compliance only at six weeks visit (n = 1) (S6 Table). Analysed group sizes are depicted in the respective result tables. Little’s Missing Completely At Random (MCRA)’s test of the entire data set indicated that values were missing at random.

### Effect of the probiotic interventions on markers of intestinal barrier function

None of the probiotic interventions significantly affected the urinary L/R ratio compared to the placebo at six weeks (primary outcome, Tables 2 and 3 for per protocol analyses). Applying an intention-to-treat approach resulted in higher p-values (single strain: p = 0.821, dual strain: p = 0.915, S7 Table), while multiple imputation of missing values resulted in slightly lower p-values (single strain: p = 0.442, dual strain: p = 0.468, S8 Table).

**Table 2:** Age- and L/R-baseline-adjusted, baseline-corrected estimated means for markers of intestinal barrier function, immune response, and stress in the *single strain* group compared to placebo after three and six weeks.

	3 weeks					6 weeks				
Marker	Placebo	Single strain	p-value	η_p_^2^	CV	Placebo	Single strain	p-value	η_p_^2^	CV
L/R	-0.024 (-0.097, 0.049) n = 16	-0.028 (-0.101, 0.045) n = 16	0.931	0.000	*L/R*	-0.006 (-0.089, 0.076) n = 16	-0.030 (-0.115, -0.055) n = 15	0.689	0.006	-
S/E	0.049 (-0.022, 0.120) n = 17	-0.020 (-0.091, 0.051) n = 17	0.175	0.061	-	0.050 (-0.051, 0.151) n = 16	-0.024 (-0.128, 0.081) n = 15	0.315	0.037	-
I-FABP (pg/mL)	-0.017 (-0.171, 0.136) n = 14	-0.064 (-0.213, 0.084) n = 15	0.658	0.008	-	0.033 (-0.078, 0.145) n = 15	-0.031 (-0.146, 0.085) n = 14	0.424	0.026	-
LBP (ng/mL)	0.011 (-0.035, 0.058) n = 14	-0.026 (-0.071, 0.019) n = 15	0.247	0.053	-	0.022 (-0.014, 0.058) n = 15	-0.012 (-0.049, 0.025) n = 14	0.191	0.067	*L/R*
VIP (ng/mL)	-0.001 (-0.034, 0.032) n = 13	0.006 (-0.026, 0.038) n = 14	0.752	0.004	*age*	-0.000 (-0.053, 0.053) n = 13	-0.006 (-0.056, 0.043) n = 15	0.863	0.001	*age*
f-Calprotectin (mg/kg)	-0.078 (-0.321, 0.164) n = 17	-0.100 (-0.335, 0.136) n = 18	0.900	0.001	-	0.001 (-0.194, 0.196) n = 16	-0.247 (-0.430, -0.063) n = 18	0.071	0.105	-
hs-CRP (mg/L)	0.079 (-0.056, 0.213) n = 14	-0.043 (-0.177, 0.092) n = 14	0.204	0.066	-	0.056 (-0.133, 0.246) n = 15	-0.094 (-0.284, 0.096) n = 15	0.266	0.047	-
IL-6 (pg/mL)	0.025 (-0.032, 0.083) n = 13	-0.030 (-0.083, 0.023) n = 15	0.159	0.081	*L/R*, *age*	**0.164** **(0.062, 0.266)** n = 15	**0.016** **(-0.090, 0.122)** n = 14	**0.052**	**0.142**	-
IL-8 (pg/mL)	-0.023 (-0.069, 0.024) n = 14	-0.007 (-0.052, 0.038) n = 15	0.628	0.010	-	0.033 (-0.039, 0.104) n = 14	0.031 (-0.041, 0.103) n = 14	0.976	0.000	-
IL-10 (pg/mL)	-0.015 (-0.069, 0.040) n = 14	-0.059 (-0.112, -0.007) n = 15	0.241	0.055	-	0.010 (-0.092, 0.112) n = 15	0.023 (-0.083, 0.128) n = 14	0.861	0.001	-
IFN-y (pg/mL)	0.048 (-0.072, 0.167) n = 14	-0.036 (-0.152, 0.080) n = 15	0.315	0.040	-	0.027 (-0.151, 0.205) n = 15	0.165 (-0.019, 0.349) n = 14	0.287	0.045	-
TNF-a (pg/mL)	0.027 (-0.005, 0.059) n = 14	0.004 (-0.027, 0.034) n = 15	0.288	0.045	-	0.014 (-0.026, 0.054) n = 15	0.013 (-0.029, 0.054) n = 14	0.962	0.000	-
IL-4 (pg/mL)	0.039 (-0.111, 0.189) n = 14	0.036 (-0.109, 0.180) n = 15	0.973	0.000	-	0.047 (-0.122, 0.216) n = 15	0.013 (-0.162, 0.189) n = 14	0.780	0.003	-
IL-5 (pg/mL)	-0.058 (-0.227, 0.110) n = 14	0.018 (-0.145, 0.180) n = 15	0.515	0.017	-	-0.037 (-0.223, 0.148) n = 15	0.140 (-0.052, 0.332) n = 14	0.191	0.067	-
CAR (AUCi, nmol/L*min)	-0.075 (-0.190, 0.041) n = 16	-0.027 (-0.135, 0.082) n = 18	0.545	0.012	-	0.003 (-0.062, 0.067) n = 16	0.013 (-0.052, 0.077) n = 16	0.825	0.002	-
Cortisol S1 (nmol/L)	-0.098 (-0.228, 0.033) n = 16	0.009 (-0.110, 0.129) n = 19	0.230	0.046	-	-0.067 (-0.145, 0.067) n = 17	-0.006 (-0.084, 0.072) n = 17	0.268	0.041	-

All biomarker data are shown as estimated marginal means of log-transformed, baseline-corrected data controlled for age and L/R baseline values using one-way analysis of covariance (ANCOVA). Confidence intervals are depicted in brackets under the estimated means. Descriptive p-values < 0.05 were considered statistically significant. η_p_^2^ –effect size (partial eta squared). CV—significant effect of covariate in ANCOVA model (p<0.05). L/R—lactulose/rhamnose excretion ratio. S/E—sucralose/erythritol excretion ratio. I-FABP—intestinal fatty acid-binding protein. LBP—lipopolysaccharide-binding protein. VIP—vasoactive intestinal polypeptide. f-Calprotectin—faecal calprotectin. hs-CRP—high sensitivity C-reactive protein. IL—interleukin. IFN-γ–interferon-gamma. TNF-α–tumour necrosis factor-alpha. CAR—Cortisol awakening response. AUCi—Area under the curve with respect to the increase. S1 –first saliva sample after awakening.

**Table 3:** Age- and L/R-baseline-adjusted, baseline-corrected estimated means for markers of intestinal barrier function, immune response, and stress in the *dual strain* group compared to placebo after three and six weeks.

	3 weeks					6 weeks				
Marker	Placebo	Dual strain	p-value	η_p_^2^	CV	Placebo	Dual strain	p-value	η_p_^2^	CV
L/R	-0.012 (-0.093, 0.070) n = 16	0.073 (-0.001, 0.147) n = 19	0.142	0.068	-	0.014 (-0.059, 0.086) n = 16	0.041 (-0.026, 0.107) n = 19	0.597	0.009	-
S/E	0.036 (-0.036, 0.108) n = 17	0.053 (-0.015, 0.121) n = 19	0.731	0.004	-	0.033 (-0.063, 0.129) n = 16	0.037 (-0.050, 0.125) n = 19	0.941	0.000	-
I-FABP (pg/mL)	0.025 (-0.158, 0.208) n = 14	-0.108 (-0.291, 0.075) n = 14	0.316	0.042	-	0.049 (-0.071, 0.168) n = 15	-0.026 (-0.130, 0.079) n = 19	0.371	0.027	-
LBP (ng/mL)	0.002 (-0.063, 0.067) n = 14	0.043 (-0.022, 0.108) n = 14	0.381	0.032	-	0.012 (-0.043, 0.067) n = 15	-0.002 (-0.051, 0.046) n = 18	0.701	0.005	-
VIP (ng/mL)	0.000 (-0.057, 0.056) n = 13	0.048 (-0.006, 0.102) n = 14	0.222	0.064	-	-0.009 (-0.068, 0.051) n = 13	-0.002 (-0.050, 0.047) n = 19	0.857	0.001	-
f-Calprotectin (mg/kg)	-0.022 (-0.305, 0.260) n = 17	-0.178 (-0.429, 0.074) n = 21	0.429	0.019	-	-0.007 (-0.192, 0.178) n = 16	-0.216 (-0.376, -0.057) n = 21	0.104	0.078	-
hs-CRP (mg/L)	0.086 (-0.098, 0.269) n = 14	-0.028 (-0.212, 0.155) n = 14	0.391	0.031	-	**0.053** **(-0.116, 0.222)** **n = 15**	**-0.194** **(-0.342, -0.045)** **n = 19**	**0.041**	**0.132**	-
IL-6 (pg/mL)	0.043 (-0.074, 0.160) n = 13	0.070 (-0.042, 0.182) n = 14	0.744	0.005	-	0.142 (0.024, 0.259) n = 15	0.060 (-0.042, 0.163) n = 19	0.320	0.033	-
IL-8 (pg/mL)	-0.013 (-0.067, 0.042) n = 14	0.016 (-0.038, 0.071) n = 14	0.458	0.023	-	0.022 (-0.065, 0.109) n = 14	0.028 (-0.044, 0.099) n = 20	0.928	0.000	-
IL-10 (pg/mL)	-0.013 (-0.071, 0.046) n = 14	-0.063 (-0.122, 0.004) n = 14	0.240	0.057	-	0.062 (-0.036, 0.159) n = 15	-0.029 (-0.113, 0.054) n = 20	0.182	0.057	-
IFN-y (pg/mL)	0.061 (-0.104, 0.226) n = 14	0.082 (-0.083, 0.246) n = 14	0.861	0.001	-	0.025 (-0.108, 0.158) n = 15	0.035 (-0.079, 0.148) n = 20	0.916	0.000	-
TNF-a (pg/mL)	0.029 (-0.022, 0.079) n = 14	-0.016 (-0.067, 0.035) n = 14	0.232	0.059	-	0.009 (-0.036, 0.055) n = 15	0.018 (-0.022, 0.058) n = 19	0.781	0.003	-
IL-4 (pg/mL)	0.054 (-0.103, 0.211) n = 14	-0.049 (-0.206, 0.108) n = 14	0.366	0.034	-	0.063 (-0.133, 0.260) n = 15	-0.001 (-0.168, 0.166) n = 20	0.632	0.008	-
IL-5 (pg/mL)	-0.075 (-0.286, 0.136) n = 14	-0.060 (-0.271, 0.151) n = 14	0.924	0.000	-	-0.041 (-0.304, 0.221) n = 15	-0.231 (-0.455, -0.007) n = 20	0.295	0.035	-
CAR (AUCi, nmol/L*min)	-0.050 (-0.163, 0.062) n = 16	0.016 (-0.081, 0.113) n = 21	0.391	0.022	-	0.003 (-0.073, 0.079) n = 16	0.013 (-0.052, 0.078) n = 20	0.846	0.001	-
Cortisol S1 (nmol/L)	-0.092 (-0.221, 0.037) n = 16	-0.115 (-0.265, 0.034) n = 21	0.818	0.002	-	-0.077 (-0.207, 0.053) n = 17	-0.045 (-0.158, 0.068) n = 22	0.720	0.004	-

All biomarker data are shown as estimated marginal means of log-transformed, baseline-corrected data controlled for age and L/R baseline values using one-way analysis of covariance (ANCOVA). Confidence intervals are depicted in brackets under the estimated means. Descriptive p-values < 0.05 were considered statistically significant. η_p_^2^ –effect size (partial eta squared). CV—significant effect of covariate in ANCOVA model (p<0.05). L/R—lactulose/rhamnose excretion ratio. S/E—sucralose/erythritol excretion ratio. I-FABP—intestinal fatty acid-binding protein. LBP—lipopolysaccharide-binding protein. VIP—vasoactive intestinal polypeptide. f-Calprotectin—faecal calprotectin. hs-CRP—high sensitivity C-reactive protein. IL—interleukin. IFN-γ–interferon-gamma. TNF-α–tumour necrosis factor-alpha. CAR—Cortisol awakening response. AUCi—Area under the curve with respect to the increase. S1 –first saliva sample after awakening.

Also, neither large intestinal permeability (assessed as urinary S/E ratio) nor intestinal epithelial cell damage (plasma I-FABP levels) were significantly affected by the interventions (Tables 2 and 3, secondary outcomes).

### Effect of the probiotic interventions on markers of immune response and stress

After correction for multiplicity, none of the secondary and exploratory markers were significantly affected. When applying descriptive p-values, serum IL-6 levels showed a tendency to be reduced in the single strain group (age- and L/R-baseline-adjusted, baseline-corrected, log-transformed mean of 0.016 pg/mL [confidence interval: -0.090, 0.122]) compared to the placebo group (placebo: 0.164 [0.062, 0.266] pg/mL, p = 0.052, Table 2) after six weeks. In the dual strain group, hsCRP values were significantly reduced compared to the placebo group after six weeks (placebo: 0.053 [-0.116, 0.222] mg/L, dual strain: -0.194 [-0.342, -0.045] mg/L, p = 0.041, Table 3). After multiple imputation of missing values, p-values for both these outcomes were not significant anymore (IL-6: p = 0.385, hsCRP: p = 0.123, S8 Table), however, 4 out of 5 imputations for hsCRP resulted in p-values < 0.05. Unadjusted results for the secondary outcomes for which age or baseline L/R showed significant effects in the ANCOVA model did not differ substantially from the adjusted values (S4 Table).

Cortisol awakening response and cortisol levels at awakening were not significantly affected by any of the probiotic interventions (Tables 2 and 3).

### Post-hoc power calculations

As the planned sample sizes of 75 was not achieved, we included a post hoc power analysis to see the effect on the study’s power. Using the means and standard values obtained in this study, for the primary outcome regarding the single strain, 15 900 individuals per group would have been needed (See S9 Table). For the dual strain, n = 48 participants per group would have been required to show a significant difference. Also for all secondary outcomes, our actual sample sizes were too small to achieve significant effects (S9 Table).

### Effect of the probiotic interventions on symptoms of IBS, anxiety and depression as well as health status—Exploratory outcomes

The single strain did not have any statistically significant effects on the symptoms assessed in this study (Table 4). After three weeks, the dual strain group showed significantly reduced total GSRS scores (placebo: 0.009 [-0.052, 0.071], dual strain: -0.085 [-0.140, -0.030], descriptive p = 0.032, Table 5) as well as GSRS-pain subscores compared to the placebo group (placebo: 0.001 [-0.077, 0.079], dual strain: -0.122 [-0.192, -0.052], p = 0.028, Table 5). These reductions were not statistically significant anymore after six weeks (Table 5). In addition, the effect on GSRS total scores at three weeks was not significant when leaving the ANCOVA model unadjusted for age (S4 Table, p = 0.253).

**Table 4:** Age- and L/R-baseline-adjusted, baseline-corrected estimated means for symptoms of IBS, anxiety and depression and quality of life in the *single strain* group compared to placebo after three and six weeks.

	3 weeks					6 weeks				
Scoring system	Placebo (n = 17)	Single strain (n = 18)	p-value	η_p_^2^	CV	Placebo (n = 17)	Single strain (n = 18)	p-value	η_p_^2^	CV
IBS-SSS	-0.096 (-0.182, -0.010)	-0.118 (-0.201, -0.035)	0.710	0.005	-	-0.109 (-0.213, -0.005)	-0.144 (-0.245, -0.043)	0.628	0.008	-
GSRS-Total	-0.025 (-0.066, -0.017)	-0.059 (-0.098, -0.019)	0.238	0.045	*L/R*, *age*	-0.086 (-0.139, -0.033)	-0.100 (-0.151, -0.048)	0.714	0.004	-
GSRS-Pain	-0.018 (-0.092, 0.055)	-0.097 (-0.168, -0.025)	0.132	0.072	-	-0.081 (-0.153, -0.010)	-0.095 (-0.165, -0.026)	0.778	0.003	-
GSRS-Bloating	-0.020 (-0.084, 0.043)	-0.091 (-0.152, -0.029)	0.119	0.076	-	-0.072 (-0.144, 0.001)	-0.130 (-0.200, -0.060)	0.252	0.042	*L/R*
GSRS-Constipation	-0.088 (-0.192, 0.016)	-0.026 (-0.127, 0.075)	0.394	0.024	-	-0.080 (-0.165, 0.006)	-0.059 (-0.142, 0.024)	0.728	0.004	-
GSRS-Diarrhoea	-0.041 (-0.093, 0.010)	-0.049 (-0.099, 0.002)	0.840	0.001	-	-0.126 (-0.204, -0.048)	-0.113 (-0.189, -0.038)	0.819	0.002	-
GSRS-Early satiety	0.018 (-0.081, 0.118)	-0.049 (-0.146, 0.047)	0.330	0.031	*L/R*,*age*	-0.075 (-0.168, 0.019)	-0.085 (-0.176, 0.006)	0.876	0.001	-
HADS-Anxiety	-0.027 (-0.117, 0.062)	-0.097 (-0.184, -0.010)	0.263	0.040	-	-0.097 (-0.190, -0.004)	-0.060 (-0.151, 0.030)	0.570	0.011	-
HADS-Depression	-0.020 (-0.087, 0.046)	-0.025 (-0.090, 0.040)	0.929	0.000	-	-0.082 (-0.159, -0.005)	-0.100 (-0.175, -0.026)	0.733	0.004	-
Health Status (EQ-VAS)	0.021 (-0.014, 0.056)	0.041 (0.007, 0.075)	0.413	0.022	-	0.000 (-0.044, 0.044)	0.025 (-0.018, 0.068)	0.428	0.020	-

All symptom data are shown as estimated marginal means of log-transformed, baseline-corrected data controlled for age and baseline L/R using one-way analysis of covariance (ANCOVA). Confidence intervals are depicted in brackets under the estimated means. Descriptive p-values < 0.05 were considered statistically significant. η_p_^2^ –effect size (partial eta squared). CV—significant effect of covariate in ANCOVA model (p<0.05). IBS-SSS—Irritable bowel syndrome-severity scoring system. GSRS—Gastrointestinal symptom scale. HADS—Hospital anxiety and depression score. EQ—EuroQol. VAS—Visual analogue scale.

**Table 5:** Age- and L/R-baseline-adjusted, baseline-corrected estimated means for symptoms of IBS, anxiety and depression and quality of life in the *dual strain* group compared to placebo after three and six weeks.

	3 weeks					6 weeks				
Scoring system	Placebo (n = 17)	Dual strain (n = 21–22)	p-value	η_p_^2^	CV	Placebo (n = 17)	Dual strain (n = 22)	p-value	η_p_^2^	CV
IBS-SSS	-0.079 (-0.163, 0.005)	-0.150 (-0.223, -0.077)	0.226	0.042	-	-0.092 (-0.189, 0.005)	-0.172 (-0.256, -0.088)	0.231	0.041	-
GSRS-Total	0.009 (-0.052, 0.071)	-0.085 (-0.140, -0.030)	**0.032**	0.129	*Age*	-0.068 (-0.124, -0.012)	-0.103 (-0.152, -0.054)	0.370	0.023	-
GSRS-Pain	0.001 (-0.077, 0.079)	-0.122 (-0.192, -0.052)	**0.028**	0.134	-	-0.061 (-0.147, 0.026)	-0.134 (-0.209, -0.059)	0.221	0.042	-
GSRS-Bloating	0.011 (-0.069, 0.091)	-0.083 (-0.155, -0.012)	0.096	0.079	*Age*	-0.057 (-0.119, 0.005)	-0.083 (-0.137, -0.029)	0.539	0.011	-
GSRS-Constipation	-0.038 (-0.163, 0.086)	-0.046 (-0.157, 0.065)	0.929	0.000	-	-0.054 (-0.155, 0.047)	-0.065 (-0.153, 0.023)	0.877	0.001	-
GSRS-Diarrhoea	-0.013 (-0.087, 0.061)	-0.090 (-0.156, -0.024)	0.143	0.062	-	-0.105 (-0179, -0.031)	-0.133 (-0.197, -0.069)	0.580	0.009	-
GSRS-Early satiety	0.076 (-0.048, 0.200)	-0.070 (-0.180, 0.041)	0.096	0.079	*Age*	-0.058 (-0.166, 0.050)	-0.079 (-0.173, 0.015)	0.777	0.002	-
HADS-Anxiety	0.000 (-0.075, 0.074)	-0.071 (-0.135, -0.006)	0.175	0.052	-	-0.065 (-0.163, 0.033)	-0.145 (-0.230, -0.060)	0.239	0.039	-
HADS-Depression	-0.015 (-0.112, 0.081)	-0.054 (-0.138, 0.030)	0.560	0.010	-	-0.062 (-0.144, 0.020)	-0.047 (-0.118, 0.025)	0.788	0.002	-
Health status (EQ-VAS)	0.021 (-0.017, 0.058)	0.006 (-0.027, 0.040)	0.579	0.009	-	0.008 (-0.036, 0.051)	0.005 (-0.033, 0.043)	0.933	0.000	-

All symptom data are shown as estimated marginal means of log-transformed, baseline-corrected data controlled for age and baseline L/R using one-way analysis of covariance (ANCOVA). Confidence intervals are depicted in brackets under the estimated means. Descriptive p-values < 0.05 were considered statistically significant. η_p_^2^ –effect size (partial eta squared). CV—significant effect of covariate in ANCOVA model (p<0.05). IBS-SSS—Irritable bowel syndrome-severity scoring system. GSRS—Gastrointestinal symptom scale. HADS—Hospital anxiety and depression score. EQ—EuroQol. VAS—Visual analogue scale.

### Effect of the probiotic interventions on stool frequency and consistency

**Fig 2:**
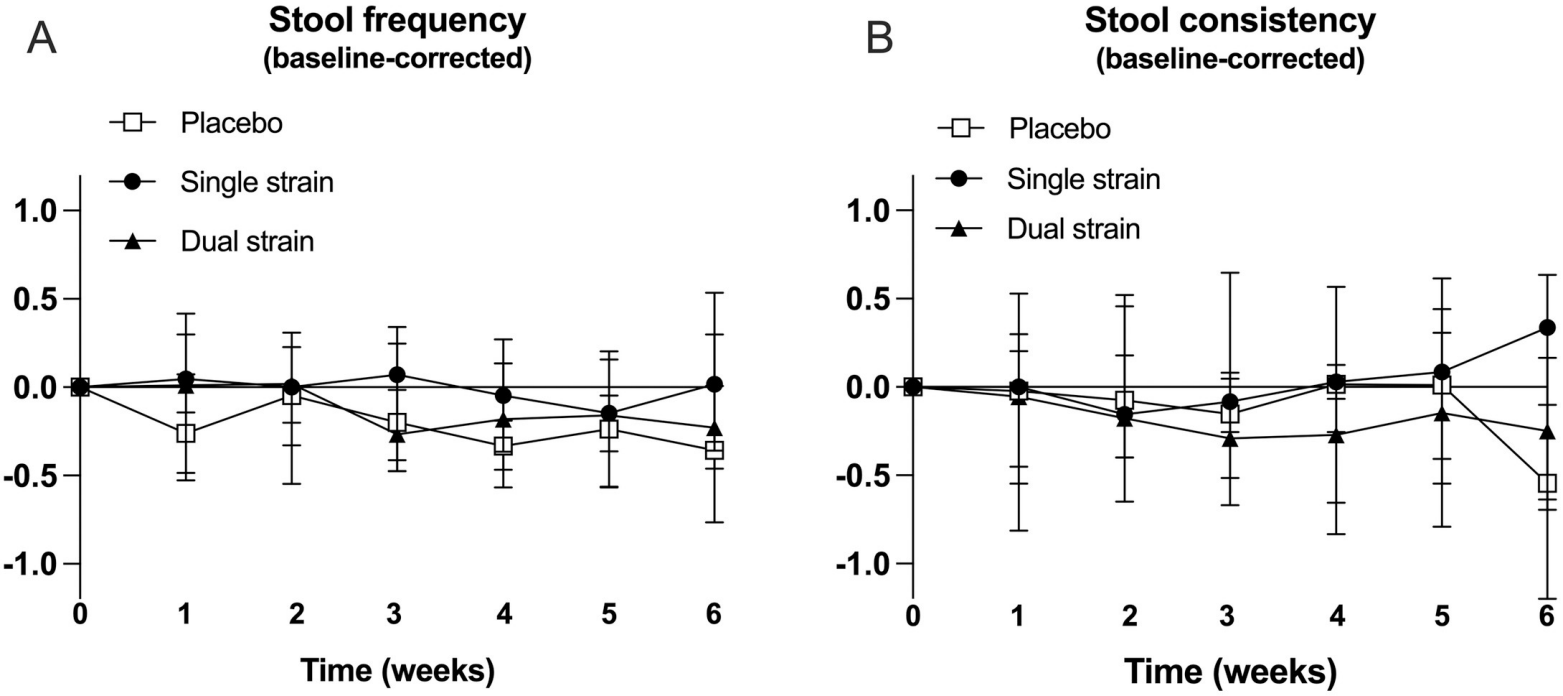
Bristol stool chart diary data. A: Baseline-corrected stool frequency (average per day). B: Baseline-corrected stool consistency (average per day).

Mixed-effect analysis of the Bristol stool chart diary data showed no significant time*group interaction effects of the interventions on stool frequency (F (12, 301) = 1.22, p = 0.270) nor consistency (F (12, 301) = 1.06, p = 0.397, Fig 2).

## Discussion

A six-week intervention with *L*. *reuteri* ATCC PTA 6475 alone (single strain) or in combination with *L*. *reuteri* DSM 17938 (dual strain) did not significantly affect intestinal barrier function in patients with IBS-D. When correcting for multiplicity, none of the secondary or exploratory outcomes were significantly affected by the interventions. Nevertheless, for hypothesis-generating purposes, we report descriptive p-values indicating decreased plasma IL-6 and hs-CRP concentrations in the single strain and dual strain group, respectively, after six weeks. In addition, the dual strain group showed significantly reduced gastrointestinal symptoms after three weeks compared to the placebo group.

Although both *L*. *reuteri* ATCC PTA 6475 and *L*. *reuteri* DSM 17938 have shown beneficial effects on intestinal barrier function *in vitro* and in mice studies [19–21], we could not see any effect on small and large intestinal permeability assessed by a urinary recovery multi-sugar test in IBS-D patients. This could be due to that we did not achieve the planned sample sizes (n = 25 per group) as a result of the Covid-19 pandemic. However, post-hoc power calculations showed that even with n = 25 per group, we would not have achieved significant results.

Both strains used in this study have shown barrier-protecting effects *in vitro* and *in vivo*. In Caco-2 cell monolayers, both strains showed a tendency to reduce intestinal permeability [21]. In a porcine intestinal cell line (IPEC-J2), they prevented an increase of intestinal permeability in cells infected with enterotoxigenic *Escherichia coli* (ETEC), possibly by maintaining E-cadherin expression and upregulating ZO1-195 [19]. Suggested mechanisms include competition with ETEC for binding to tight junction proteins and blocking of the proinflammatory response [19]. In addition, both strains reduced intestinal injury as a result of cow milk and lipopolysaccharide exposure in newborn rats [20], which was partly attributed to their anti-inflammatory properties [20].

It is not uncommon that properties of probiotics tested *in vitro* or in mice fail to translate to humans [49]. Nevertheless, other probiotic strains have been able to improve barrier function even in clinical studies. A mix of different probiotics (*Streptococcus thermophilus*, *Lactobacillus bulgaricus*, *L*. *acidophilus*, *Bifidobacterium longum*) was able to reduce intestinal permeability measured as the urinary lactulose/mannitol (L/M) ratio in patients with IBS-D after 4 weeks of intervention [50]. Also, an 8-week intervention with *Lacticaseibacillus rhamnosus* GG improved symptoms and intestinal permeability (L/M ratio) in children with IBS or functional pain [51]. In a recent open-label study with IBS-D patients, a multi-strain mix reduced small intestinal permeability which was assessed using radionuclide tracers [52]. On the other hand, *Bacillus subtilis* BS50 and *Bifidobacterium breve* Bif195 did not significantly affect intestinal permeability in healthy adults [53] or in healthy trained adults after intense exercise [54], respectively. These opposing results may be due to strain-specific effects of the probiotics investigated or differences in study design.

We assessed intestinal permeability using the non-invasive urinary multi-sugar recovery test as well as surrogate markers in blood (such as I-FABP). Even if the multi-sugar test is a rather sensitive and site-specific method, it can be affected by post-absorptive factors [55], as well as being dependent on the participants’ compliance regarding dietary restrictions and duration of collection. It is possible that applying more direct methods, such as investigating the permeability of intestinal biopsies using, e.g., the Ussing chamber or immunohistochemical staining, would have yielded different results, and future studies should consider this.

Interestingly, both the single and the dual strain showed potential anti-inflammatory effects in this study (single strain: reduced IL-6 levels, dual strain: reduced hsCRP levels). Both strains have been shown to reduce inflammatory markers, including IL-6, in several *in vitro* and mice studies [20, 25, 56–58]. While *L*. *reuteri* ATCC PTA 6475 has shown to inhibit secretion of cytokines involved in the inflammatory response such as tumour necrosis factor (TNF)-α, interleukin (IL)-6 and IL-1β through histamine receptor H2 [22–25], *L*. *reuteri* DSM 17938 was shown to be capable of inducing regulatory T cells by means of adenosine A_2A_ receptor activation [26–29]. *L*. *reuteri* ATCC PTA 6475 has also been shown to form biofilms with immune-modulatory properties [57] and to modulate intestinal dendritic cells towards an anti-inflammatory phenotype [58].

In clinical studies, *L*. *reuteri* ATCC PTA 6475 by itself has so far been investigated in older women with low bone mineral density [59]. This study showed improvement in bone mineral density but found no differences in inflammatory markers. In a subsequent analysis of the same study population, the beneficial outcome on bone mineral density was linked to reduction of hsCRP [60]. The anti-inflammatory effect of the strain combination has been investigated in humans with regards to its effect on *Helicobacter pylori* eradication therapy [32, 61], and recently also in children with autism [62] as well as IBS [30]. In children with autism, intake of the dual strain for six months was able to improve social function, but had no clear effects on the children’s immune profiles [62]. In the IBS study, which included all IBS subtypes, faecal calprotectin levels were significantly reduced by the dual strain and in a similar magnitude as seen in our study, even if our study did not find statistical significance (Table 3, p = 0.104 for the dual strain, p = 0.071 for the single strain). These potential anti-inflammatory properties warrant further investigation in future larger studies, possibly also in other disease cohorts where low-grade inflammation plays a role (e.g., metabolic syndrome, depressive disorders).

With regards to effects on gastrointestinal symptoms, the dual strain seemed to significantly improve total GSRS scores as well as pain subscores. However, these effects were statistically significant only after three weeks. The dual strain also showed a tendency to reduce symptoms of bloating after three weeks (dual strain: p = 0.096, Table 5). However, due to small group sizes and p-values being descriptive, these results should be interpreted with caution.

In previous studies, *L*. *reuteri* DSM 17938 strain by itself has been shown to reduce pain in children with functional abdominal pain or IBS [63–65] and adults [66], as well as to improve symptoms and bowel movement frequency in adults with functional constipation [67, 68]. The recently published study in IBS patients including all IBS subtypes showed that the same combination of strains, however in a slightly lower dose, combined with standard care, significantly improved IBS symptoms [30]. This improvement of symptoms started at six weeks of intervention and lasted until the end of the study (14 weeks), suggesting that the intervention period in our study might have been too short. Improvements in symptoms were related to abdominal pain, pain relieved by bowel action, bloating, passing gas, and visible distension. In addition, quality of life and anxiety scores were significantly improved, which was not the case in our study. Similar to our study, there was no statistically significant effect on stool consistency evaluated by the Bristol stool scale. Noticeably, the study by Cruchet et al. had a larger sample size (n = 70 per group), which might explain the higher number of significant findings in their study. Also, all subtypes of IBS were included in their study, and it could be that the efficacy of the probiotic intervention is dependent on the predominant symptom and, e.g., less effective in reducing symptoms in the diarrhoea subtype used in the current study.

Our study comes with several limitations. Due to the COVID-19 pandemic, the number of study participants was lower than anticipated which resulted in limited power, and after correction for multiplicity, none of the results were significant. In general, sample sizes were too small to achieve significant effects. Hence, findings should be interpreted with caution. Also, median age differed between the groups, and even if data was baseline-corrected and analysed using ANCOVA with age as a covariate, this could have still affected the results, especially as levels of inflammatory markers can be age-dependent. Future studies should also consider a mixed model approach as a possible methodological alternative. Furthermore, small intestinal permeability at baseline was lower in the dual strain group compared to the single strain (p = 0.038) and the placebo group (p = 0.130), which could have also affected the results. Future studies should consider a more elaborate randomisation process, including factors such as age and main outcomes such as intestinal permeability. In addition, a fourth arm with only *L*. *reuteri* DSM 17938 might have allowed comparisons of efficacy regarding the dual strain versus the respective single strains. Moreover, especially with regards to the results of the recent study by Cruchet et al., the intervention period of six weeks might have been too short to observe significant results [30].

In conclusion, a 6-week intervention with *L*. *reuteri* ATCC PTA 6475 alone or in combination with *L*. *reuteri* DSM 17938 did not improve intestinal permeability or barrier function in patients with IBS-D. However, a possible positive effect on immune markers and gastrointestinal symptoms provides incentives for future, larger-scale studies.

## Supporting information

S1 Table CONSORT checklist. (DOC)

S2 Table Exclusion criteria. (DOCX)

S3 Table Protocol deviations. (DOCX)

S4 Table Unadjusted results. (DOCX)

S5 Table Adverse events. (DOCX)

S6 Table Missing values overview. (DOCX)

S7 Table Intention to treat analysis. (DOCX)

S8 Table Multiple imputation. (DOCX)

S9 Table Post hoc power analyses. (DOCX)

S1 File (PDF)

S2 File (PDF)
