# Correction: The effects of a 6-week intervention with *Limosilactobacillus reuteri* ATCC PTA 6475 alone and in combination with *L*. *reuteri* DSM 17938 on gut barrier function, immune markers, and symptoms in patients with IBS-D—An exploratory RCT

**DOI:** 10.1371/journal.pone.0316404

**Published:** 2025-02-21

**Authors:** The PLOS ONE Editors

## Notice of Republication

This article was republished on December 3, 2024, to address an issue identified post-publication. Please download this article again to view the correct version.

The article’s Data Availability statement is updated to: The data are pseudonymised, meaning that the key variable may not be destroyed. According to Swedish ethics regulations, the raw data cannot be shared without an approved ethics application from the National Swedish Ethics Authority. An ethical permit can only be obtained for research being conducted within Sweden. Data requests can be emailed to forskningsdata@oru.se.
