# Supplementary material for: The effects of a 6-week intervention with Limosilactobacillus reuteri ATCC PTA 6475 alone and in combination with L. reuteri DSM 17938 on gut barrier function, immune markers, and symptoms in patients with IBS-D—An exploratory RCT
Source: PLoS One. 2024 Nov 1;19(11):e0312464. doi: 10.1371/journal.pone.0312464 (PMC11530048; doi:10.1371/journal.pone.0312464)
Supplement: S2 Table — (DOCX) [file pone.0312464.s002.docx]

**S2 Table. Exclusion criteria**

1. Known organic gastrointestinal disease (e.g., inflammatory bowel disease)
2. Previous abdominal surgery which might influence gastrointestinal function, except appendectomy and cholecystectomy
3. History of or present gastrointestinal malignancy or polyposis
4. Recently (within the last six months) diagnosed gastrointestinal infection
5. Current diagnosis of dementia, severe depression, major psychiatric disorder, or other incapacity for adequate cooperation
6. Chronic neurological/neurodegenerative diseases (e.g., Parkinson’s disease, multiple sclerosis)
7. Autoimmune disease and/or patients receiving immunosuppressive medication
8. Chronic pain syndromes (e.g., fibromyalgia)
9. Chronic fatigue syndrome
10. Severe endometriosis
11. Coeliac disease
12. Recently (within the last three months) diagnosed lactose intolerance
13. Pregnant or breastfeeding
14. Regular intake of systemic corticosteroids and anti-inflammatory medication (including non-steroidal anti-inflammatory drugs) during the last three months or incidental use in the last two weeks prior to randomisation
15. Recent (<four weeks prior to randomisation) intake of proton pump inhibitors (e.g., omeprazole)
16. Use of anti-depressants in the last three months
17. Regular oral intake of mast cell stabilising drugs (e.g., sodium cromoglycate) during the last three months or incidental use in the last two weeks prior to screening
18. Antimicrobial treatment six weeks prior to first screening visit
19. Antimicrobial prophylaxis (e.g., acne, urinary tract infection)
20. Regular consumption of probiotic products four weeks prior to first baseline visit
21. Concurrent or recent (<four weeks prior to randomisation) use of nutritional supplements or herb products affecting intestinal function (e.g., aloe vera, St. John’s Wort, fibres, prebiotics) if the investigator considers those could affect the study outcome
22. Inability to maintain exercise routine and dietary pattern during the study
23. Abuse of alcohol or drugs
24. Any clinically significant present or past disease/condition which in the investigator’s opinion could interfere with the results of the trial
