# Supplementary material for: The effects of a 6-week intervention with Limosilactobacillus reuteri ATCC PTA 6475 alone and in combination with L. reuteri DSM 17938 on gut barrier function, immune markers, and symptoms in patients with IBS-D—An exploratory RCT
Source: PLoS One. 2024 Nov 1;19(11):e0312464. doi: 10.1371/journal.pone.0312464 (PMC11530048; doi:10.1371/journal.pone.0312464)
Supplement: S3 Table — (DOCX) [file pone.0312464.s003.docx]

|  | **Placebo** | **Single Strain** | **Dual Strain** |
| --- | --- | --- | --- |
| **Type of violation** |  |  |  |
| Compliance <80% | n=1: all samples | n=1: all samples  n=1: all 6 week samples | n=1: all samples |
| Intake of NSAID close to or during sampling | n=1, baseline 2 visit: all urine and blood samples excluded, faecal samples and saliva OK (collected before) | n=1, week 3 visit: all urine and blood samples excluded, faecal samples and saliva OK (collected before) | n=1, week 3&6 visit: all urine samples excluded, blood, faecal samples and saliva OK (collected before) |
| Intake of antiviral medication close to or during sampling | n=1, week 3 visit: all saliva samples excluded, other samples OK |  |  |
| Delayed sampling | - | n=1, week 6 visit: all urine and blood samples excluded, faecal samples and saliva collected on time  n=1, week 6 visit: all urine and saliva samples excluded, blood and faecal samples collected on time | - |

**S3 Table: Excluded values due to protocol violations**

NSAID – non-steroidal anti-inflammatory drugs.
