# Supplementary material for: The effects of a 6-week intervention with Limosilactobacillus reuteri ATCC PTA 6475 alone and in combination with L. reuteri DSM 17938 on gut barrier function, immune markers, and symptoms in patients with IBS-D—An exploratory RCT
Source: PLoS One. 2024 Nov 1;19(11):e0312464. doi: 10.1371/journal.pone.0312464 (PMC11530048; doi:10.1371/journal.pone.0312464)
Supplement: S4 Table — (DOCX) [file pone.0312464.s004.docx]

**S4 Table: Baseline-corrected estimated means of ANCOVA**

| **Unadjusted for *‘L/R baseline’* (only *‘age’* as covariate)** | | | | |
| --- | --- | --- | --- | --- |
| **Marker** | **Placebo** | **Probiotic** | **p-value** | **η_p_^2^** |
| L/R, 3w, single strain | -0.012  (-0.089, 0.065) | -0.041  (-0.118, 0.036) | 0.589 | 0.010 |
| IL-6, 3w, single strain | 0.017  (-0.046, 0.079) | -0.023  (-0.081, 0.036) | 0.354 | 0.034 |
| LBP, 6w, single strain | 0.016  (-0.022, 0.054) | -0.006  (-0.045, 0.033) | 0.405 | 0.027 |
| GSRS-Total, 3w, single strain | -0.016  (-0.061, 0.029) | -0.066  (-0.110, -0.022) | 0.116 | 0.076 |
| GSRS-Early satiety, 3w, single strain | 0.035  (-0.071, 0.140) | -0.065  (-0.167, 0.038) | 0.177 | 0.056 |
| GSRS-Bloating, 6w, single strain | -0.062  (-0.137, 0.013) | -0.139  (-0.213, -0.066) | 0.143 | 0.066 |
|  | | | | |
| **Unadjusted for *‘age‘* (only *‘L/R baseline’* as covariate)** | | | | |
| **Marker** | **Placebo** | **Probiotic** | **p-value** | **η_p_^2^** |
| VIP, 3w, single strain | -0.004  (-0.041, 0.033) | 0.008  (-0.027, 0.044) | 0.628 | 0.010 |
| VIP, 6w, single strain | -0.002  (-0.060, 0.055) | -0.004  (-0.057, 0.049) | 0.964 | 0.000 |
| IL-6, 3w, single strain | 0.028  (-0.034, 0.090) | -0.032  (-0.090, 0.025) | 0.159 | 0.078 |
| GSRS-Total, 3w, single strain | -0.023  (-0.070, 0.023) | -0.060  (-0.105, -0.015) | 0.260 | 0.039 |
| GSRS-Total, 3w, dual strain | -0.015  (-0.080, 0.049) | -0.065  (-0.123, -0.007) | 0.253 | 0.037 |
| GSRS-Bloating, 3w, dual strain | -0.014  (-0.094, 0.067) | -0.063  (-0.136, 0.009) | 0.359 | 0.024 |
| GSRS-Early satiety, 3w, single strain | 0.022  (-0.090, 0.133) | -0.052  (-0.160, 0.056) | 0.334 | 0.028 |
| GSRS-Early satiety, 3w, dual strain | 0.035  (-0.091, 0.161) | -0.036  (-0.149, 0.077) | 0.399 | 0.020 |
|  | | | | |
| **Unadjusted for *‘age‘* and *‘L/R baseline’* (ANOVA)** | | | | |
| **Marker** | **Placebo** | **Probiotic** | **p-value** | **η_p_^2^** |
| IL-6, 3w, single strain | 0.020  (-0.044, 0.085) | -0.026  (-0.086, 0.034) | 0.295 | 0.042 |
| GSRS-Total, 3w, single strain | -0.017  (-0.065, 0.032) | -0.066  (-0.113, -0.019) | 0.143 | 0.064 |
| GSRS-Early satiety, 3w, single strain | 0.034  (-0.079, 0.147) | -0.064  (-0.174, 0.046) | 0.212 | 0.047 |

All biomarker data are shown as estimated marginal means of log-transformed, baseline-corrected data using one-way analysis of covariance (ANCOVA). Confidence intervals are depicted in brackets under the estimated means. Descriptive p-values < 0.05 were considered statistically significant. η_p_^2^ – effect size (partial eta squared). L/R – lactulose/rhamnose excretion ratio. IL – interleukin. LBP – lipopolysaccharide-binding protein. VIP - vasoactive intestinal polypeptide. GSRS – Gastrointestinal symptoms rating scale. 3w – 3 weeks follow-up visit. 6w – 6 weeks follow-up visit.
