# Supplementary material for: The effects of a 6-week intervention with Limosilactobacillus reuteri ATCC PTA 6475 alone and in combination with L. reuteri DSM 17938 on gut barrier function, immune markers, and symptoms in patients with IBS-D—An exploratory RCT
Source: PLoS One. 2024 Nov 1;19(11):e0312464. doi: 10.1371/journal.pone.0312464 (PMC11530048; doi:10.1371/journal.pone.0312464)
Supplement: S5 Table — (DOCX) [file pone.0312464.s005.docx]

**S5 Table:** **Adverse events after start of intervention**.

|  | **Placebo**  **(n=17)** | **Single strain**  **(n=19)** | **Dual strain (n=22)** |
| --- | --- | --- | --- |
| Total events | 12 | 14 | 7 |
| Abdominal pain | 2 | 4 | 0 |
| Asthma | 1 | 1 | 0 |
| Bloating | 1 | 2 | 1 |
| Diarrhoea | 0 | 1 | 1 |
| Elective minor surgery | 0 | 1 | 0 |
| Fungal vaginosis | 1 | 0 | 0 |
| Gastroenteritis | 1 | 2 | 0 |
| Headache | 0 | 1 | 1 |
| Household accident | 1 | 0 | 0 |
| Upper airway infection | 5 | 2 | 4 |

No significant differences were found between the groups (Fisher’s exact test).
