# Supplementary material for: The effects of a 6-week intervention with Limosilactobacillus reuteri ATCC PTA 6475 alone and in combination with L. reuteri DSM 17938 on gut barrier function, immune markers, and symptoms in patients with IBS-D—An exploratory RCT
Source: PLoS One. 2024 Nov 1;19(11):e0312464. doi: 10.1371/journal.pone.0312464 (PMC11530048; doi:10.1371/journal.pone.0312464)
Supplement: S6 Table — (DOCX) [file pone.0312464.s006.docx]

|  | **3 weeks** | | | **6 weeks** | | |
| --- | --- | --- | --- | --- | --- | --- |
|  | **Placebo**  (n=17) | **Single strain**  (n=19) | **Dual strain**  (n=22) | **Placebo**  (n=17) | **Single strain**  (n=19) | **Dual strain**  (n=22) |
| **Outcome** | **% (n) missing values** | | | **% (n) missing values** | | |
| L/R | 5.9 (1) | 15.8 (3) | 13.6 (3) | 5.9 (1) | 21.1 (4) | 13.6 (3) |
| S/E | 0.0 (0) | 10.5 (2) | 13.6 (3) | 5.9 (1) | 21.1 (4) | 13.6 (3) |
| I-FABP | 17.6 (3) | 21.1 (4) | 36.4 (8) | 11.8 (2) | 26.3 (5) | 13.6 (3) |
| LBP | 17.6 (3) | 21.1 (4) | 36.4 (8) | 11.8 (2) | 26.3 (5) | 13.6 (3) |
| VIP | 23.5 (4) | 26.3 (5) | 36.4 (8) | 23.5 (4) | 21.1 (4) | 13.6 (3) |
| f-Calpro | 0.0 (0) | 5.3 (1) | 4.5 (1) | 5.9 (1) | 5.3 (1) | 4.5 (1) |
| hsCRP | 17.6 (3) | 26.3 (5) | 36.4 (8) | 11.8 (2) | 21.1 (4) | 13.6 (3) |
| IL-6 | 23.5 (4) | 21.1 (4) | 36.4 (8) | 11.8 (2) | 26.3 (5) | 13.6 (3) |
| IL-8 | 17.6 (3) | 21.1 (4) | 36.4 (8) | 17.6 (3) | 26.3 (5) | 9.1 (2) |
| IL-10 | 17.6 (3) | 21.1 (4) | 36.4 (8) | 11.8 (2) | 26.3 (5) | 9.1 (2) |
| TNF-a | 17.6 (3) | 21.1 (4) | 36.4 (8) | 11.8 (2) | 26.3 (5) | 13.6 (3) |
| IFN-y | 17.6 (3) | 21.1 (4) | 36.4 (8) | 11.8 (2) | 26.3 (5) | 9.1 (2) |
| IL-4 | 17.6 (3) | 21.1 (4) | 36.4 (8) | 11.8 (2) | 26.3 (5) | 9.1 (2) |
| IL-5 | 17.6 (3) | 21.1 (4) | 36.4 (8) | 11.8 (2) | 26.3 (5) | 9.1 (2) |
| CAR (AUCi) | 5.9 (1) | 5.3 (1) | 4.5 (1) | 5.9 (1) | 15.8 (3) | 4.5 (1) |
| Cortisol S1 | 5.9 (1) | 0.0 (0) | 4.5 (1) | 0.0 (0) | 10.5 (2) | 0.0 (0) |
| IBS-SSS | 0.0 (0) | 5.3 (1) | 0.0 (0) | 0.0 (0) | 5.3 (1) | 0.0 (0) |
| GSRS-IBS | 0.0 (0) | 5.3 (1) | 0.0 (0) | 0.0 (0) | 5.3 (1) | 4.5 (1) |
| HADS | 0.0 (0) | 5.3 (1) | 0.0 (0) | 0.0 (0) | 5.3 (1) | 0.0 (0) |
| Health score | 0.0 (0) | 5.3 (1) | 5.3 (1) | 0.0 (0) | 5.3 (1) | 0.0 (0) |

**S6 Table: Overview of missing values**

This table shows all missing values, e.g., due to cancelled study visits (covid-19), protocol violations, outliers, technical errors. L/R – lactulose/rhamnose excretion ratio. S/E – sucralose/erythritol excretion ratio. I-FABP – intestinal fatty acid-binding protein. LBP – lipopolysaccharide-binding protein. VIP - vasoactive intestinal polypeptide. f-Calpro – faecal calprotectin. hs-CRP – high sensitivity C-reactive protein. IL – interleukin. IFN-γ – interferon-gamma. TNF-α – tumour necrosis factor-alpha. CAR – Cortisol awakening response. AUCi – Area under the curve with respect to the increase. S1 – first saliva sample after awakening. IBS-SSS – Irritable bowel syndrome-severity scoring system. GSRS-IBS – Gastrointestinal symptom scale IBS version. HADS – Hospital anxiety and depression score.
