# Supplementary material for: The effects of a 6-week intervention with Limosilactobacillus reuteri ATCC PTA 6475 alone and in combination with L. reuteri DSM 17938 on gut barrier function, immune markers, and symptoms in patients with IBS-D—An exploratory RCT
Source: PLoS One. 2024 Nov 1;19(11):e0312464. doi: 10.1371/journal.pone.0312464 (PMC11530048; doi:10.1371/journal.pone.0312464)
Supplement: S7 Table — (DOCX) [file pone.0312464.s007.docx]

**S7Table: Intention to treat analysis of primary outcome**

| **Marker** | **Placebo** | **Single strain** | **p-value** | **η_p_^2^** | **CV** | **Placebo** | **Dual strain** | **p-value** | **η_p_^2^** | **CV** |
| --- | --- | --- | --- | --- | --- | --- | --- | --- | --- | --- |
| L/R, 6w | 0.006  (-0.093, 0.105)  n=19 | 0.022  (-0.077, 0.121)  n=19 | 0.821 | 0.002 | - | 0.035  (-0.042, 0.111)  n=19 | 0.041  (-0.032, 0.133)  n=21 | 0.915 | 0.000 | - |

Data are shown as estimated marginal means of log-transformed, baseline-corrected data controlled for age and L/R baseline values using one-way analysis of covariance (ANCOVA). Confidence intervals are depicted in brackets under the estimated means. P-values < 0.05 were considered statistically significant. L/R – lactulose/rhamnose excretion ratio. η_p_^2^ – effect size. CV – significant effect of covariate in ANCOVA model.
