# Supplementary material for: The effects of a 6-week intervention with Limosilactobacillus reuteri ATCC PTA 6475 alone and in combination with L. reuteri DSM 17938 on gut barrier function, immune markers, and symptoms in patients with IBS-D—An exploratory RCT
Source: PLoS One. 2024 Nov 1;19(11):e0312464. doi: 10.1371/journal.pone.0312464 (PMC11530048; doi:10.1371/journal.pone.0312464)
Supplement: S8 Table — (DOCX) [file pone.0312464.s008.docx]

**S8 Table: Age- and L/R-baseline-adjusted, baseline-corrected estimated means after multiple imputation of missing values for main outcomes (6-week follow-up)**

|  | **Placebo** | **Single strain** | **p-value** | **η_p_^2^** |
| --- | --- | --- | --- | --- |
| **L/R** | 0.013 (-0.291, 0.316) | 0.078 (-0.185, 0.342) | 0.442 | 0.031 |
| **IL-6** | 0.157 (0.016, 0.297) | 0.060 (-0.144, 0.263) | 0.385 | 0.052 |
|  |  |  |  |  |
|  | **Placebo** | **Dual strain** | **p-value** | **η_p_^2^** |
| **L/R** | 0.052 (-0.352, 0.430) | 0.059 (-0.269, 0.386) | 0.468 | 0.019 |
| **hsCRP** | 0.063 (-0.132, 0.258) | -0.177 (-0.402, 0.047) | 0.123 | 0.143 |

All data are shown as estimated marginal means of log-transformed, baseline-corrected data controlled for age and L/R baseline values using one-way analysis of covariance (ANCOVA) after multiple imputation of missing values. Multiple imputation was conducted using the automatic method in SPSS, generating five imputed datasets. Pooled results of all five imputations are shown. Confidence intervals are depicted in brackets next to the estimated means. η_p_^2^ – effect size (partial eta squared). L/R – lactulose/rhamnose excretion ratio. IL – interleukin. hs-CRP – high sensitivity C-reactive protein.
