# Supplementary material for: The effects of a 6-week intervention with Limosilactobacillus reuteri ATCC PTA 6475 alone and in combination with L. reuteri DSM 17938 on gut barrier function, immune markers, and symptoms in patients with IBS-D—An exploratory RCT
Source: PLoS One. 2024 Nov 1;19(11):e0312464. doi: 10.1371/journal.pone.0312464 (PMC11530048; doi:10.1371/journal.pone.0312464)
Supplement: S9 Table — (DOCX) [file pone.0312464.s009.docx]

|  | **Single strain** | | **Dual strain** | |
| --- | --- | --- | --- | --- |
| **Outcome** | **Required sample size^1^ (n)** | **Observed power^2^ (%)** | **Required sample size^1^ (n)** | **Observed power^2^ (%)** |
| L/R | 15 901 | 5.1 | 48 | 40.7 |
| S/E | 176 | 12.8 | 179 | 14.6 |
| I-FABP (pg/mL) | 976 | 6.3 | 158 | 15.2 |
| LBP (ng/mL) | 83 | 20.7 | 182 | 13.8 |
| VIP (ng/mL) | 712 | 6.7 | 2008 | 5.7 |
| f-Calprotectin (mg/kg) | 67 | 28.8 | 80 | 27.6 |
| hs-CRP (mg/L) | 935 | 6.5 | 69 | 28.3 |
| IL-6 (pg/mL) | 47 | 33.2 | 52 | 35.4 |
| IL-8 (pg/mL) | 580 | 7.0 | 533 | 7.6 |
| IL-10 (pg/mL) | 312 | 8.9 | 233 | 10.5 |
| IFN-y (pg/mL) | 38 | 37.7 | 354 | 9.2 |
| TNF-a (pg/mL) | 230 | 10.5 | 901 | 6.8 |
| IL-4 (pg/mL) | 307 | 9.1 | 86 | 22.2 |
| IL-5 (pg/mL) | 168 | 11.9 | 72 | 28.7 |

**S9 Table: Post hoc power analyses**

^1^Post hoc power analyses were performed using the actual mean values and standard deviations obtained in the study, based on a power of 80%, and a significance (alpha) level of 5% with two-sided testing. ^2^Observed power was calculated using the actual sample sizes achieved in the study, assuming a power of 80%, and a significance (alpha) level of 5% with two-sided testing. L/R – lactulose/rhamnose excretion ratio. S/E – sucralose/erythritol excretion ratio. I-FABP – intestinal fatty acid-binding protein. LBP – lipopolysaccharide-binding protein. VIP - vasoactive intestinal polypeptide. f-Calprotectin – faecal calprotectin. hs-CRP – high sensitivity C-reactive protein. IL – interleukin. IFN-γ – interferon-gamma. TNF-α – tumour necrosis factor-alpha.
