# Supplementary material for: The effects of a 6-week intervention with Limosilactobacillus reuteri ATCC PTA 6475 alone and in combination with L. reuteri DSM 17938 on gut barrier function, immune markers, and symptoms in patients with IBS-D—An exploratory RCT
Source: PLoS One. 2024 Nov 1;19(11):e0312464. doi: 10.1371/journal.pone.0312464 (PMC11530048; doi:10.1371/journal.pone.0312464)
Supplement: S2 File — (PDF) [file pone.0312464.s011.pdf]

CLINICAL STUDY PROTOCOL

The effect of two probiotic products on the intestinal barrier function in patients with irritable bowel syndrome

PRINCIPAL INVESTIGATOR
Professor Robert JM Brummer, MD, PhD
Örebro University
701 82 Örebro
Tel: +46 19 30 37 31
E-mail: robert.brummer@oru.se

Date of Protocol: March 24, 2022

Version 2

Table of Contents

1 STUDY GROUP AND STRUCTURE 4

2 BACKGROUND INFORMATION 5

2.1 IRRITABLE BOWEL SYNDROME 5
2.2 INTESTINAL PERMEABILITY IN IBS 5
2.3 ASSESSMENT OF INTESTINAL BARRIER FUNCTION 6
2.4 PROBIOTIC INTERVENTION STUDIES 7

3 STUDY OBJECTIVES 7

4 STUDY DESIGN 8

4.1 STUDY PERIOD 9

5 STUDY POPULATION 9

5.1 NUMBER OF SUBJECTS 9
5.2 RECRUITMENT 10
5.3 INCLUSION AND EXCLUSION CRITERIA FOR IBS-D PATIENTS 10

6 METHODS 11

6.1 SCREENING PROCEDURES 11
6.2 CASE REPORT FORMS (CRF) 12
6.3 INVESTIGATIONAL PRODUCT 12
6.4 RANDOMISATION 12
6.5 SYMPTOM SCALES 13
6.5.1 DAILY DIARY ABOUT STOOL FREQUENCY AND CONSISTENCY 13
6.5.2 IBS - SEVERITY SCORING SYSTEM (IBS-SSS) 13
6.5.3 GASTROINTESTINAL SYMPTOM RATING SCALE - IBS (GSRS-IBS) 13
6.5.4 QUALITY OF LIFE SCALE FOR IBS PATIENTS (IBS-QOL) 13
6.5.5 HEALTH-RELATED QUALITY OF LIFE (EUROQOL EQ-5D-5L) 13
6.5.6 HOSPITAL ANXIETY AND DEPRESSION SCALE (HADS) 13
6.5.7 PERCEIVED STRESS SCALE (PSS) 14
6.6 PERMEABILITY ASSAY 14
6.7 COLLECTION OF BLOOD 14
6.8 CORTISOL AWAKENING RHYTHM (SALIVA) 14
6.9 COLLECTION OF FAECAL SAMPLES 15

7 CONCOMITANT TREATMENT 15

8 RESTRICTIONS AND INSTRUCTIONS FOR STUDY PARTICIPANTS 15

9 SAFETY AND ETHICAL CONSIDERATIONS 16

10 WITHDRAWAL CRITERIA 16

11 ADVERSE EVENTS 16

12 DATA MANAGEMENT AND STATISTICAL ANALYSIS 17

13 INSURANCE 17

14 COMPENSATION OF STUDY PARTICIPANTS 17

15 REFERENCES 18

APPENDIX I: ROME IV CRITERIA 21

APPENDIX II: QUESTIONNAIRE ON BACKGROUND INFORMATION 22

APPENDIX III: FOOD FREQUENCY QUESTIONNAIRE (FFQ) 26

APPENDIX IV: 3-DAY FOOD DIARY 27

APPENDIX V: DIARY ABOUT STOOL FREQUENCY AND CONSISTENCY 29

APPENDIX VI: IBS-SSS 30

APPENDIX VII: GSRS-IBS 31

APPENDIX VIII: IBS-QOL 32

APPENDIX IX: 5Q-5D-5L 34

APPENDIX X: HADS 38

APPENDIX XI: PERCEIVED STRESS SCALE (PSS) 39

1 Study group and structure

Study centre

Örebro University
Nutrition-Gut-Brain Interactions Research Centre
Faculty of Health and Medicine, School of Medical Sciences
701 82 Örebro, Sweden

Sponsor and principal investigator

Prof. Robert JM Brummer, MD, PhD

University Hospital Örebro
Department of Gastroenterology
701 85 Örebro

phone: 0046 (0) 19 - 602 20 84

Örebro University
Nutrition-Gut-Brain Interactions (NGBI)
Research Centre
School of Medical Sciences
701 82 Örebro

phone: 0046 (0) 19 - 30 37 31
email: robert.brummer@oru.se

Project leader

Julia König

Örebro University
School of Medical Sciences
701 82 Örebro

phone: 0046 (0) 19 - 30 3645

email: julia.konig@oru.se

Participating investigators/List of responsible parties

Julia König, PhD, NGBI Research Centre, School of Medical Sciences, Örebro University

María Fernanda Roca Rubio, PhD student, NGBI Research Centre, School of Medical Sciences, Örebro University

Hanna Edebol-Carlman, PhD, NGBI Research Centre, School of Medical Sciences, Örebro University

Jakob Axelsson, PhD, BioGaia

2 Background information

2.1 Irritable bowel syndrome

Irritable bowel syndrome (IBS) is a very common disorder with a worldwide prevalence of 10-20%. Even though it is not life-threatening or associated with higher mortality, it profoundly affects the patients' quality of life and causes substantial economic costs due to the need for medical consultation and work absenteeism (Sandler et al. 2002).
Symptoms vary between patients and include constipation and/or diarrhoea, abdominal pain and cramps, flatulence, faecal urgency, a sense of incomplete evacuation and relief of pain or discomfort upon defaecation. IBS is classified according to the Rome IV criteria as recurrent abdominal pain, on average, at least 1 day/week in the last 3 months associated with 2 or more of the following: Related to defaecation, associated with a change in frequency of stool, or associated with a change in form (appearance) of stool (Lacy 2015). IBS subtypes can further be classified according to predominant stool patterns: IBS with constipation (IBS-C), IBS with diarrhoea (IBS-D), and IBS with alternating symptoms of diarrhoea and constipation (IBS-M), and unsubtyped IBS (Longstreth et al. 2006).
Even though the aetiology and pathophysiology of IBS are complex and not well understood, it is well accepted that a dysregulation of the microbe-gut-brain axis plays an important role. Associated aberrations include visceral hypersensitivity, abnormal gut motility, gut microbiota changes and autonomic nervous system dysfunction (Mayer et al. 2015). In addition, there is a growing amount of data revealing a contributing role of an aberrant immune system in the pathogenesis of IBS. Mild immune activation has been found both locally in the gut and systemically (Barbara et al. 2011; Sundin et al. 2014). Furthermore, psychological and environmental factors like anxiety, depression and significant negative life events are believed to contribute to IBS development (Mayer et al. 2015).

Diagnosis of IBS is mainly based on symptomatology and absence of clear organic diseases, rather than on pathophysiologic mechanisms. There are no preventive strategies and current therapy is not firmly based on sound physiologic principles but mainly aims at alleviating symptoms of abdominal pain or altered bowel transit. The efficacy of current treatment is limited and new therapies are needed.

2.2 Intestinal permeability in IBS

The intestinal barrier is an important component in the interplay between the gut and the brain. It prevents translocation of harmful substances and pathogens from the external environment while allowing a peaceful coexistence with intestinal symbionts without causing chronic inflammation (König et al. 2016). The physical barrier is mainly formed by the mucus layer, the epithelial layer and the underlying lamina propria. Tight junction proteins connect the intestinal epithelial cells and regulate paracellular permeability. The functional barrier includes components such as immune cells, the intestinal microbiota and antimicrobial peptides, which play crucial roles in supporting appropriate gut barrier function. Disruption of this barrier results in increased intestinal permeability, which in turn facilitates translocation of harmful substances and

pathogens to the bloodstream (König et al. 2016). An increased intestinal permeability has been related to several diseases such as inflammatory bowel diseases, coeliac disease, obesity, and also irritable bowel syndrome (IBS) (König et al. 2016). Studies using *in vivo* methods have shown that about 40% of IBS patients, and mostly those with diarrhoea as their main symptom, have signs of increased intestinal permeability (Dunlop et al. 2006) (Zhou, Zhang, and Verne 2009) (Zhou et al. 2010) (Gecse et al. 2012) (Mujagic et al. 2014). *Ex vivo* experiments showed that colon biopsies of IBS patients display an increased permeability and release soluble factors that can reduce barrier function in other cells (Piche et al. 2009) (Han et al. 2012). Also psychological stress, which is often increased in IBS patients, is known to enhance intestinal permeability via the hypothalamic-pituitary-adrenal (HPA) axis and mast cell activation (Vanuytsel et al. 2014).

2.3 Assessment of intestinal barrier function

The permeability of the small intestine is commonly evaluated by measurement of the intestinal permeation and urinary excretion of orally administered water-soluble, non-fermentable sugars that differ in size. The larger sugar molecules, such as lactulose, can only cross the intestinal barrier by paracellular passage, and are not taken up actively. The smaller molecules, such as rhamnose, cross the epithelial barrier transcellularly and act as a control for gastric emptying, dilution, transit time, and epithelial absorptive area, as well as systemic distribution and renal function. The urinary excretion ratio is then used as a standardised assessment of the intestinal permeability of the intestinal segment where the permeability probes are absorbed (König et al. 2016; van Wijck et al. 2013).

Other potential biomarkers of epithelial integrity, which have a causal relationship with permeability and innate barrier function, are molecules produced by epithelial cells. Serum levels of tight junction proteins such as zonulin are promising biomarkers of epithelial integrity (Wells et al. 2017). Zonulin can also be detected in faecal samples.

Additional sensitive biomarkers for detection of tissue injury are fatty acid-binding proteins (FABP), which are tissue-specific, small cytosolic proteins (Pelsers, Hermens, and Glatz 2005). Intestinal FABP (i-FABP) is particularly highly expressed in cells present on the tips of the villi and not in the crypt (Pelsers et al. 2003). As the villi is the initial site of destruction, upon epithelial damage i-FABP proteins are released into the systemic circulation, and have therefore been reported as useful markers to detect early stages of epithelial cell damage (Wells et al. 2017). i-FABP can be measured in plasma using enzyme-linked immunosorbent assays (ELISAs) (Wells et al. 2017).

An increase in intestinal permeability potentially leads to translocation of microbiota or their toxic products. A promising markers reflecting translocation of bacteria or their products into the circulation is bacterial 16S ribosomal RNA (rRNA).

The cortisol awakening rhythm is a robust marker of stress and has been assessed in several clinical disorders, including IBS. For example, Suárez-Hitz et al. found that IBS patients with predominant diarrhea exhibited substantially heightened cortisol levels at awakening and a blunted cortisol awakening response (Suarez-Hitz et al. 2012).

2.4 Probiotic intervention studies

Several meta-analyses have shown that the intake of specific beneficial bacteria, so-called probiotics, can improve gastrointestinal symptoms of IBS patients (Moayyedi et al. 2010; Ford et al. 2014). In addition, there is evidence that administration of probiotics and prebiotics can positively affect stress levels (Pinto-Sanchez et al. 2017) (Takada et al. 2016) (Schmidt et al. 2015). The mechanisms behind the beneficial effect of probiotics are still mostly unknown. A possible mechanism could be a strengthening of the barrier function. A study by principal investigator Robert Brummer showed that a probiotic administered by a feeding tube could strengthen the intestinal barrier both in healthy volunteers as well as IBS patients (Karczewski et al. 2010). Both strains in the probiotic products used in the current study have shown to reduce intestinal permeability in pre-clinical studies (Karimi et al. 2018).

3 Study objectives

The overarching aim of this study is to investigate the effect of a 6-week intervention with two different probiotic products ((*Lactobacillus reuteri* ATCC PTA 6475 och Gastrus (*Lactobacillus reuteri* DSM 17938/ATCC PTA 6475)) on the intestinal permeability of diarrhoea-predominant IBS (IBS-D) patients.

The primary endpoint is the difference in small intestinal permeability, measured as the urinary lactulose/rhamnose secretion ratio, after a 6-week intervention with the probiotic products compared to the placebo product, in IBS-D patients.

Secondary endpoints are the differences between intervention with the probiotic products compared to the placebo in IBS-D patients on:

- The small intestinal permeability, measured as the urinary lactulose/rhamnose secretion ratio, after 3-week intervention
- Whole gut permeability measured as urinary sucralose/erythritol excretion ratio (0-24hrs) after 3 and 6 weeks
- Colonic permeability measured as urinary sucralose/erythritol ratio (5-24hrs) after 3 and 6 weeks
- Gastroduodenal permeability measured as urinary sucrose excretion (0-5hrs) after 3 and 6 weeks
- Various potential biomarkers of general intestinal barrier function and inflammation in blood and faecal samples after 3 and 6 weeks such as plasma i-FABP, faecal zonulin, serum zonulin, bacterial 16S ribosomal RNA (16S rRNA) gene copy number (whole blood), plasma vasoactive intestinal polypeptide (VIP), plasma high-sensitive C-reactive protein (hs-CRP), faecal calprotectin and plasma cytokines (e.g. IL-6 and IL-10)

Exploratory endpoints are the effects of the intervention with the probiotic products in comparison to the placebo in IBS-D patients on:

- Stool frequency and consistency (Daily diary on bowel movement using Bristol Stool Chart)
- Gastrointestinal symptoms as measured by the GSRS-IBS and IBS-SSS after 3 and 6 weeks
- Quality of life after 3 and 6 weeks (5Q-5D-5L, IBS-QOL)
- Perceived stress after 6 weeks (using the Perceived stress scale, PSS)
- Anxiety and depression symptoms after 3 and 6 weeks (HADS)
- Cortisol awakening rhythm (saliva) after 3 and 6 weeks
- Intestinal microbiota composition (16S rRNA-based next generation sequencing of faecal samples) after 3 and 6 weeks
- Metabolomic profile of faecal samples and blood, including SCFA metabolism and tryptophan catabolites such as 5-HT and melatonin, after 3 and 6 weeks.

In addition, we want to use the data from the baseline visits to investigate the correlations between the assessed biomarkers in urine, blood, saliva and faeces as well as with the different symptom scales. Also, intra- and interindividual variations of these markers will be investigated. Data will also be analysed separated by sex and with regards to patient characteristics such as BMI and age.

4 Study design

This will be a single-centre, randomised, double-blinded, placebo-controlled intervention study with a parallel design and three study arms.

The study will investigate the effects of supplementation with the probiotic products *L. reuteri* ATCC PTA 6475 and Gastrus (*L. reuteri* DSM 17938/ATCC PTA 6475) on the intestinal barrier function, gut health and stress symptoms in diarrhoea-predominant IBS (IBS-D) patients. The test product is a tablet containing either ATCC PTA 6475 or Gastrus (DSM 17938/ATCC PTA 6475). The subjects will consume the product twice a day, giving a daily dose of 1 x 10^9 CFU of each strain. The placebo product is an identical tablet except for the absence of probiotics. After having given their written informed consent, subjects will complete screening procedures to evaluate their eligibility for the study (visit 1). Study participants will undergo two baseline visits (visits 2 and 3, 3 weeks apart) to account for individual variations, and will then be randomly assigned to 6-week intake of either ATCC PTA 6475, Gastrus, or placebo in a ratio of 1:1:1 (stratified by sex). Participants will attend a study visit three weeks into the intervention (visit 4) and at the end of the intervention (visit 5, week 6). The intestinal permeability will be assessed using a standardized multi-sugar test at visit 2, visit 3, visit 4, and at end of intervention (visit 5). Blood, saliva and faecal samples will be collected at visit 2-5. In addition, gastrointestinal symptoms will be recorded at those visits (using GSRS-IBS, IBS-SSS). Quality of life using the 5Q-5D-5L and Hospital Anxiety and Depression Scale (HADS) scores will also be recorded at those visits. In addition, at visit 3 and visit 5, the IBS-QoL and the Perceived Stress Scale (PSS) will be completed. In a daily diary, participants will record stool frequency and consistency from visit 2 until end-of-study visit 5. Dietary habits of the patients will be assessed via a food frequency questionnaire (FFQ) and a 3-day food diary before the intervention (between visit 2 and 3).

During the entire run-in and intervention period, the subjects will be asked not to consume any probiotic products other than the study products. During the entire study subjects should maintain their habitual diet and life style such as physical activity level and sleep habits.

Table 1: Study flow chart

	Screening	*Baseline*		*Intervention*	
Assessment	*Visit 1 before-3w*	*Visit 2 -3w*	*Visit 3 0w*	*Visit 4 3w*	*Visit 5 6w*
	**Screening**	**Baseline 1**	**Baseline 2**	**Follow-up 1**	**Follow-up 2**
Screening	**x**				
Permeability test (urine)		x	x	x	x
Blood sampling		x	x	x	x
Faecal sample collection		x	x	x	x
Cortisol (saliva)		x	x	x	x
IBS symptom scales (GSRS-IBS, IBS-SSS)		x	x	x	x
Daily diary - stool frequency and consistency		x			
Quality of life (5Q-5D-5L)		x	x	x	x
Quality of life (QoL-IBS)			x		x
Hospital anxiety and depression scale (HADS)		x	x	x	x
Perceived stress scale (PSS)			x		x
Food Frequency Questionnaire		x			
3-day food diary		x			
Compliance diary				x	
Adverse events reporting				x	

4.1 Study Period

It is anticipated that the study will start during the first quarter of 2019 and recruitment is anticipated to be complete within 12 months of start.

5 Study population

5.1 Number of subjects

The sample size is based on the ability to find a significant lactulose/rhamnose ratio difference between one of the probiotic study arms and the placebo arm. A relevant change of 15% in lactulose/rhamnose difference should be detected as significant, which according to the study by Zuhl et al. 2014 amounts to 0.009 points in ratio difference.
The sample size calculation is based on the following assumptions:

- 80% power

- An expected SD of 0.01 (Zuhl et al. 2014)
- Significance (alpha) level of 5%
- Two-sided testing (significance level of 5%)

To account for a potential drop-out rate of 15%, a total of 75 subjects will be randomized (N=25 subjects in each group should be included).

5.2 Recruitment

Patients suffering from IBS and meeting the inclusion criteria (see 5.3) will be recruited during routine visits at the Department of Gastroenterology at the Örebro University Hospital (USÖ) and at the general health centres associated with USÖ, or through information letters sent out by mail and email to patients already diagnosed with IBS. Patients contacted by mail or email will be given the contact details of the investigators or will be asked to send in their contact details so that the investigators can contact them. In addition, advertisement for the study will be distributed at Örebro University in addition to advertisements in the local newspaper and social media. Students at Örebro University will be informed about the study via internal mailing lists, the online education platform 'blackboard' and social media. A webpage will be set up where interested participants can sign up. Interested participants will be invited to a visit at the university or the hospital. During this visit the study staff will inform carefully about the study as well as provide an informed consent form. Participants recruited during the routine visit will also be provided with a written information letter. During the visit the patient will have the opportunity to ask questions. The participants will then be given time to think through a possible participation and will be asked to sign the informed consent. If needed, the participants will be given more time to consider a participation in the study before signing the informed consent. During this time the participant will be given contact information of members of the research group in case any questions arise.

5.3 Inclusion and exclusion criteria for IBS-D patients

Inclusion criteria for patients

1. Signed informed consent
2. Fulfilled Rome IV diagnostic criteria for IBS with predominant diarrhoea
3. Mild-to-severe IBS symptoms according to the IBS severity scoring system (IBS-SSS; a score of ≥75)
4. Age: 18-65 years

Exclusion criteria for patients

1. Known organic gastrointestinal disease (e.g. IBD)
2. Previous abdominal surgery which might influence gastrointestinal function, except appendectomy and cholecystectomy
3. History of or present gastrointestinal malignancy or polyposis
4. Recently (within the last 6 months) diagnosed gastrointestinal infection

5. Current diagnosis of dementia, severe depression, major psychiatric disorder, or other incapacity for adequate cooperation
6. Chronic neurological/neurodegenerative diseases (e.g. Parkinson's disease, multiple sclerosis)
7. Autoimmune disease and/or patients receiving immunosuppressive medications
8. Chronic pain syndromes (e.g. fibromyalgia)
9. Chronic fatigue syndrome
10. Severe endometriosis
11. Coeliac disease
12. Recently (within the last 3 months) diagnosed lactose intolerance
13. Females who are pregnant or breast-feeding
14. Regular intake of systemic corticosteroids and anti-inflammatory medication (including NSAIDs) during the last 3 months or incidental use in the last 2 weeks prior to randomisation
15. Recent (< 4 weeks) intake of proton pump inhibitors, PPI (e.g., omeprazol)
16. Use of anti-depressants in the last 3 months
17. Regular oral intake of mast cell stabilising drugs (e.g. sodium cromoglycate) during the last 3 months or incidental use in the last 2 weeks prior to screening
18. Antimicrobial treatment 6 weeks prior to first screening visit
19. Antimicrobial prophylaxis (eg. acne, urinary tract infection)
20. Regular consumption of probiotic products 4 weeks prior to first baseline visit
21. Concurrent or recent (<4 weeks) use of nutritional supplements or herb products affecting intestinal function (e.g. aloe vera, St. John´s Wort, fibres, prebiotics) if the investigator considers those could affect the study outcome.
22. Inability to maintain exercise routine and dietary pattern during the study.
23. Abuse of alcohol or drugs
24. Any clinically significant present or past disease/condition which in the investigator's opinion could interfere with the results of the trial

6 Methods

6.1 Screening procedures

Informed consent

Patients must sign a dated informed consent prior to any trial-related activities. All subjects will be provided with a copy of their own signed and dated informed consent form.

Inclusion and exclusion criteria

IBS-D diagnosis will be assessed based on the Rome IV criteria by the investigator (Appendix I) and using the IBS-SSS. If not previously performed, tests to exclude other diseases such as IBD or coeliac disease will be ordered, in accordance with the Swedish IBS guidelines. Only IBS-D patients with an IBS-SSS score ≥75 will be included. Medical history, concomitant illnesses and other background information (including date of birth, sex, body weight and height, education

level, diet, current smoking habits, frequency of alcohol consumption) will be collected during a structured interview (Appendix II).

Dietary intake

Dietary intake will be assessed before and after the intervention by a food frequency questionnaire (FFQ, Appendix III) that has been validated in a Swedish population (Christensen et al. 2013) (Christensen et al. 2014). The questionnaire includes 174 foods and drinks and estimates the dietary pattern during one year. It will take approximately 20 min to complete the questionnaire. In addition, participants will be asked to record their food intake for three consecutive days using a 3-day food diary (Appendix IV).

6.2 Case Report Forms (CRF)

For each study participant data from the visits and relative parameters will be recorded on special data collection forms called Case Report Form (CRF). Each CRF must be completed and signed by the investigators to confirm that it has been completed correctly and that it is an accurate representation of the subject's participation in the study.

6.3 Investigational product

6475 tablets: $5x10^8$ CFU *Lactobacillus reuteri* ATCC PTA 6475
Gastrus tablets: $5x10^8$ CFU *Lactobacillus reuteri* DSM 17938 + $5x10^8$ CFU *Lactobacillus reuteri* ATCC PTA 6475.
Placebo tablets: Similar in shape and taste to Gastrus tablets but without the Lactobacillus reuteri components.
Both study products are delivered in identical containers.
Dosing: One tablet twice a day.
The Study Products shall be kept refrigerated at all times during the study both by the investigators and by the participants. BioGaia AB will provide the Study Products.

6.4 Randomisation

Randomisation will be computerised by an independent researcher. A third party not otherwise involved in the study will label the active and placebo study products. Randomisation lists will be kept in sealed envelopes at the study sites and a sealed copy will also be kept at Sponsor site. The envelopes may not be open until all study data have been entered into the database and until the database has been locked. For exceptions, see “Serious Adverse Event” below. Randomisation will be stratified by gender. At screening, subjects will be assigned a screening number starting at 1001, according to their chronological entry into the study. As an example the first subject will have screening number 1001. If the subjects are suitable for study participation, they will receive their randomization number at Visit 1. Randomisation numbers will include the stratification number (Female = 5; Male = 9), and will be allocated sequentially in the order in which the

subjects finalise Visit 1. For example, the first female included will receive the randomization number: 5001, whereas the fifth male included will receive the randomisation number: 9005.

Subjects will be asked to return the used packages from the Study Products to allow assessment by the investigator of compliance. The investigators will also assess compliance by a daily checklist distributed to the participants and by interviews at every visit.

6.5 Symptom scales

6.5.1 Daily diary about stool frequency and consistency

The participants will complete a daily diary about their stool frequency and consistency based on the Bristol Stool Chart (Appendix V).

6.5.2 IBS - severity scoring system (IBS-SSS)

This scale developed by Francis et al. is an additional tool to assess IBS symptoms. It evaluates five aspects of IBS during a 10-day period: abdominal pain, distension, stool frequency and consistency and interference with daily life (Appendix VI). Each item is scored on a visual analogue scale from 0 to 100 and the sum is the total score. The scale is responsive to treatment and has good validity, and takes about 5 min to complete (Drossman et al. 2011).

6.5.3 Gastrointestinal Symptom Rating Scale - IBS (GSRS-IBS)

The GSRS-IBS a reliable, valid IBS-specific symptom scale (Wiklund et al. 2003). It includes 13 items in five symptom clusters: abdominal pain, bloating, constipation, diarrhea and satiety (Appendix VII). It measures symptoms during the past 7 days with a 7-point Likert scale ranging from 1 (no discomfort at all) to 7 (very severe discomfort), and takes about 5 min to complete.

6.5.4 Quality of life scale for IBS patients (IBS-QOL)

This 26-item questionnaire developed by Wong et al. includes the following areas: bowel symptoms, fatigue, activity limitations and emotional dysfunction (Wong et al. 1998) (Appendix VIII).

6.5.5 Health-related quality of life (EuroQol EQ-5D-5L)

This is a standardized measure of health status developed by the EuroQol Group in order to provide a simple, generic measure of health for clinical and economic use (The EuroQol 1990) (Brooks 1996) (Janssen et al. 2012). The questionnaire is applicable to a wide range of health conditions and treatments. It consists of 5 items related to wellbeing and function and provides a simple descriptive profile and a single index value for health status (Appendix IX). In addition, the instrument consists of a scale where the study subject can rate her or his own experienced health status.

6.5.6 Hospital anxiety and depression scale (HADS)

This scale was originally developed by Snaith and Zigmond for use in a medical setting, and its validity and reliability has been reported in several studies (Snaith and Zigmond 1986). It consists of 14 items divided into two subscales for anxiety (seven items) and depression (seven items) (Appendix X). Patients rate each item on a four-point scale. A cut-off of > 11 implies definite

cases of anxiety or depression, a cut-off of 8–10 a probable case, and ≤ 7 no case. It takes about 5 min to complete the questionnaire.

6.5.7 Perceived stress scale (PSS)

This scale is the most widely used psychological instrument for measuring the perception of stress (Appendix XI). It is a measure of the degree to which situations in one´s life are appraised as stressful. Items were designed to tap how unpredictable, uncontrollable and overloaded the respondents find their lives. The scale also includes a number of items about current levels of experienced stress. PSS demonstrate high reliability (i.e., Cronbachs alpha = 0.84-0.86; test-retest reliability *r* = 0.85) and the scale correlates adequately with for example other stress measures, self-reported health and health behavior measures (Cohen, Kamarck, and Mermelstein 1983). This scale takes approximately 5 minutes to fill out.

6.6 Permeability assay

At visit 2-5, the intestinal permeability will be measured using a standardised multi-sugar test, which is a sensitive, non-invasive technique able to detect small changes in small and large intestinal permeability. For this procedure, 150 ml of tap water containing 1 g sucrose, lactulose, sucralose, erythritol and 0.5 g rhamnose will be orally administered after an overnight fast, followed by total urinary collection for 24 hours. During the first five hours of urine collection, the participants will refrain from food intake, and will be asked to drink at least 1.5 litres of provided water. Urine will be collected by the subjects in the provided urine bottles (3L, brown with yellow lid) and stored in cooling bags. After finalizing the five hours urine collection, subjects will continue to collect their urine for 19 hours in the provided urine bottles stored in the provided cooling bags. The study participants will return the urine samples to the university staff the following day. See chapter 8 for dietary restrictions for the participants before and during sample collection.
The urine samples will be centrifuged, aliquoted and stored at -80°C until further analysis by High Performance Liquid Chromatography (HPLC) to detect urinary excretion ratios of the sugars.

6.7 Collection of blood

Blood will be collected from each subject at visit 2-5 for analysis of the markers related to intestinal permeability and/or inflammation such as intestinal fatty acid binding protein (i-FABP), zonulin, bacterial 16S ribosomal RNA (16S rRNA) gene copy number, vasoactive intestinal polypeptide (VIP), and cytokines. Plasma, serum and whole blood will be aliquoted into cryogenic vials and frozen.

6.8 Cortisol awakening rhythm (saliva)

For the cortisol awakening rhythm, subjects will be instructed to collect saliva samples at home for three consecutive days before visits 2-5. Participants will collect the samples using a salivette directly after awakening as well as 15, 30, 45, and 60 minutes thereafter. Subjects are free to use an alarm clock or to wake up spontaneously. Subjects will be instructed not to brush their teeth before completing saliva sampling to avoid contamination of saliva with blood caused by micro-

injuries in the oral cavity. Subjects will be asked to refrain from food intake and beverages containing alcohol, caffeine or fruit juices as well as smoking during the sampling period. Besides these restrictions, subjects will be free to follow their normal morning routines on the sampling day.

6.9 Collection of faecal samples

For analysis of the faecal microbiota composition, faecal samples will be collected at visit 2-5. Subjects will collect faecal samples at home using provided faecal samples tubes (using a spoon inside the tube). The samples will be aliquoted into four vials and immediately placed into the home freezer by the subjects, and will be returned frozen to the study unit using special cool transport containers. At the study unit the faecal samples will be stored at -80°C. Faecal samples will be analysed for inflammation and barrier function markers such as faecal calprotectin and zonulin. In addition, faecal samples will be used for microbiota composition analysis (16S rRNA-based next generation sequencing, NGS) and metabolomics (microbial metabolites profile).

7 Concomitant treatment

Participants will be instructed not to take NSAIDs (e.g. ibuprofen, aspirin), anticholinergic drugs, steroids, antibiotics or antimicrobial medication, oral mast cell stabilising drugs e.g. sodium cromoglycate, and pre- and probiotic products during the study.
Any other medication, if considered necessary for the patient (e.g. (laxatives, antidiarrheals), with a stable dose (when possible) can be used but should be restricted to a minimal dose. Any treatment ongoing before the study and changes in its doses will be recorded in the CRF. In addition, subjects will record any treatment prescribed during the study.

8 Restrictions and instructions for study participants

The subjects will be asked to avoid changes in their diet and life style, such as training and sleep habits, during the study. Intake of probiotic products, as well as food and food supplements containing probiotics are not allowed from 4 weeks before the screening visit until the end of the study. Subjects will not be withdrawn from the study due to single violations, but violations will be recorded as protocol deviations. Any use of illicit drugs (euphorics or stimulants, such as cannabis, opium) is prohibited during the study. Subjects will be carefully instructed to:

- Refrain from taking any NSAIDs, antibiotics, anticholinergic drugs, oral mast cell stabilising drugs, and pre- and probiotics during the study period.
- Refrain from consuming alcohol two days prior each visit and during the 24h urine collection.
- Refrain from consuming nicotin two days prior each visit and during the 24h urine collection.
- Refrain from consuming spicy food two days prior to each visit and during the 24h urine collection.
- Refrain from consuming the artificial sugars sucralose, erythritol, lactulose and rhamnose (contained in the sugar mix) two days prior to each visit and during the 24h urine collection.
- Avoid strenuous exercise two days prior to each visit and during the 24h urine collection.

- Have the same meal in the evening prior to each visit.
- Refrain from consuming caffeine the day of each visit and during the 24h urine collection.
- The subjects shall drink at least 1.5 litres of provided water and fast (no food intake) during the first 5 hours of urine collection.

9 Safety and ethical considerations

The probiotic compounds will be studied in volunteer IBS patients, and participants will be informed about potential risks and that participation in the study may be discontinued at any time. Probiotics are known to have health-beneficial effects on gut functioning, however, mechanisms behind possible benefits from probiotics remains uncertain and thus more mechanistic studies can be justified.

Collection of faecal samples, urine and saliva is not painful or risky for the patients but it could be bothersome for some of the participants. Collection of blood will be performed by experienced nurses according to hospital routines and could in some cases cause discomfort, pain, swelling and bruising.

Participation in the study is voluntary and all subjects have the right to withdraw from the study at any time without affecting their normal treatment. Prior to any study-related activity, the study staff will give the subject oral and written information about the study. A voluntary, signed and dated informed consent form will be obtained from the patients prior to any study-related activity. This study will be conducted according to good clinical practice and the ethics of the Helsinki declaration.

Written approval will be obtained from the Central Ethical Review Board of Uppsala prior to commencement of the study.

10 Withdrawal criteria

The subject may withdraw at any time. The subject may be withdrawn from the trial by investigator or sponsor due to clinical or other reasons, if necessary. The subjects who meet any of the following criteria must be withdrawn from the study at the time of confirmation:

1. Non-IBS gastrointestinal disease in need for medical care.
2. Subject undergoing gastrointestinal surgery during the study.
3. Pregnancy.

In case of withdrawal, the withdrawal form in the CRF must be completed. Randomized subjects who do not complete the trial will be replaced (proof-of-concept study using a per-protocol analysis).

11 Adverse events

Any adverse events and adverse effects will be recorded on the CRF by the investigators. The diagnosis, if available, or each sign or symptom should be recorded. All adverse events observed must be recorded separately. Onset day and resolution day will be recorded for each event.

The investigator will assess each event with regard to its severity:

- Mild: Transient symptoms, no interference with the subject´s daily activities.

- Moderate: Marked symptoms, moderate interference with the subject´s daily activities but still acceptable.
- Severe: Considerable interference with the subjects daily activities, unacceptable.

The investigator will assess the causality of the FMT for each adverse event in the following manner:

- Unrelated: FMT has not been performed yet.
- Unlikely: The event is most likely related to an aetiology other than the FMT.
- Possible: A causal relationship is conceivable and cannot be dismissed.
- Probable: Good reason and sufficient documentation to assume a causal relationship.

Any pharmacological treatments used for the event will be recorded.

12 Data management and statistical analysis

The investigators will ensure that the subject's anonymity will be maintained. On all documents, subjects will be identified only by a subject number – not by their names or personal number.

All statistical tests will be assessed using a nominal two-sided significance level of 5%.

The primary efficacy analysis will be based on difference in lactulose/rhamnose ratio before and after intervention for the two probiotic strains as compared to placebo. The difference between before and after intervention observed in either of the two probiotic strains will be compared to that difference observed for the placebo arm of the study using the two-sided Mann-Whitney U-test for independent samples (as in Zuhl et al. 2014 and similar studies).

Biomarker levels studied in plasma, serum and stool samples will be evaluated on log-scale, where appropriate, using the appropriate non-parametric univariate tests (e.g. Wilcoxon signed-rank test, Mann-Whitney U-test), as well as multivariate analyses using appropriate cross-validation approaches (as detailed in the SAP). Questionnaires will be evaluated using both logistic regression analyses of responder proportions as well as ANOVA for original response values. Microbiota phylogenetic profiles (NGS results) will be assessed by both uni- and multivariate analyses on the species- and genus-like phylogenetic levels, as well as on in-depth functional levels based on whole genome shotgun sequencing (NGS results).

13 Insurance

All subjects will be insured by the Sponsor against injury caused by their participation in the study according to local legal requirements in Sweden where the study takes place.

14 Compensation of study participants

Based on our model for economical compensation to study subjects, participants in study will be paid 2000 Swedish kronor for compensation of discomfort and time (taxable income). Travelling

expenses to study visits will be compensated according to the public transportation rate after the study.

15 References

Barbara, G., C. Cremon, G. Carini, L. Bellacosa, L. Zecchi, R. De Giorgio, R. Corinaldesi, and V. Stanghellini. 2011. 'The immune system in irritable bowel syndrome', *Journal of neurogastroenterology and motility*, 17: 349-59.

Brooks, R. 1996. 'EuroQol: the current state of play', *Health policy (Amsterdam, Netherlands)*, 37: 53-72.

Christensen, S. E., E. Moller, S. E. Bonn, A. Ploner, O. Balter, L. Lissner, and K. Balter. 2014. 'Relative validity of micronutrient and fiber intake assessed with two new interactive meal- and Web-based food frequency questionnaires', *J Med Internet Res*, 16: e59.

Christensen, S. E., E. Moller, S. E. Bonn, A. Ploner, A. Wright, A. Sjolander, O. Balter, L. Lissner, and K. Balter. 2013. 'Two new meal- and web-based interactive food frequency questionnaires: validation of energy and macronutrient intake', *J Med Internet Res*, 15: e109.

Cohen, S., T. Kamarck, and R. Mermelstein. 1983. 'A global measure of perceived stress', *J Health Soc Behav*, 24: 385-96.

Drossman, D. A., L. Chang, N. Bellamy, H. E. Gallo-Torres, A. Lembo, F. Mearin, N. J. Norton, and P. Whorwell. 2011. 'Severity in irritable bowel syndrome: a Rome Foundation Working Team report', *The American Journal of Gastroenterology*, 106: 1749-59; quiz 60.

Dunlop, S. P., J. Hebden, E. Campbell, J. Naesdal, L. Olbe, A. C. Perkins, and R. C. Spiller. 2006. 'Abnormal intestinal permeability in subgroups of diarrhea-predominant irritable bowel syndromes', *Am J Gastroenterol*, 101: 1288-94.

Ford, A. C., E. M. Quigley, B. E. Lacy, A. J. Lembo, Y. A. Saito, L. R. Schiller, E. E. Soffer, B. M. Spiegel, and P. Moayyedi. 2014. 'Efficacy of prebiotics, probiotics, and synbiotics in irritable bowel syndrome and chronic idiopathic constipation: systematic review and meta-analysis', *Am J Gastroenterol*, 109: 1547-61; quiz 46, 62.

Gecse, K., R. Roka, T. Sera, A. Rosztoczy, A. Annahazi, F. Izbeki, F. Nagy, T. Molnar, Z. Szepes, L. Pavics, L. Bueno, and T. Wittmann. 2012. 'Leaky gut in patients with diarrhea-predominant irritable bowel syndrome and inactive ulcerative colitis', *Digestion*, 85: 40-6.

Han, W., X. Lu, X. Jia, T. Zhou, and C. Guo. 2012. 'Soluble mediators released from PI-IBS patients' colon induced alteration of mast cell: involvement of reactive oxygen species', *Digestive diseases and sciences*, 57: 311-19.

Janssen, M. F., A. S. Pickard, D. Golicki, C. Gudex, M. Niewada, L. Scalone, P. Swinburn, and J. Busschbach. 2012. 'Measurement properties of the EQ-5D-5L compared to the EQ-5D-3L across eight patient groups: a multi-country study', *Quality of life research : an international journal of quality of life aspects of treatment, care and rehabilitation*.

Karczewski, J., F. J. Troost, I. Konings, J. Dekker, M. Kleerebezem, R. J. Brummer, and J. M. Wells. 2010. 'Regulation of human epithelial tight junction proteins by Lactobacillus plantarum in vivo and protective effects on the epithelial barrier', *American journal of physiology.Gastrointestinal and liver physiology*, 298: G851-9.

Karimi, S., H. Jonsson, T. Lundh, and S. Roos. 2018. 'Lactobacillus reuteri strains protect epithelial barrier integrity of IPEC-J2 monolayers from the detrimental effect of enterotoxigenic Escherichia coli', *Physiol Rep*, 6.

König, J., J. Wells, P. D. Cani, C. L. Garcia-Rodenas, T. MacDonald, A. Mercenier, J. Whyte, F. Troost, and R. J. Brummer. 2016. 'Human Intestinal Barrier Function in Health and Disease', *Clin Transl Gastroenterol*, 7: e196.

Lacy, B. E. 2015. 'The Science, Evidence, and Practice of Dietary Interventions in Irritable Bowel Syndrome', *Clin Gastroenterol Hepatol*, 13: 1899-906.

Longstreth, G. F., W. G. Thompson, W. D. Chey, L. A. Houghton, F. Mearin, and R. C. Spiller. 2006. 'Functional bowel disorders', *Gastroenterology*, 130: 1480-91.

Mayer, E. A., J. S. Labus, K. Tillisch, S. W. Cole, and P. Baldi. 2015. 'Towards a systems view of IBS', *Nature reviews.Gastroenterology & hepatology*, 12: 592-605.

Moayyedi, P., A. C. Ford, N. J. Talley, F. Cremonini, A. E. Foxx-Orenstein, L. J. Brandt, and E. M. Quigley. 2010. 'The efficacy of probiotics in the treatment of irritable bowel syndrome: a systematic review', *Gut*, 59: 325-32.

Mujagic, Z., S. Ludidi, D. Keszthelyi, M. A. Hesselink, J. W. Kruimel, K. Lenaerts, N. M. Hanssen, J. M. Conchillo, D. M. Jonkers, and A. A. Masclee. 2014. 'Small intestinal permeability is increased in diarrhoea predominant IBS, while alterations in gastroduodenal permeability in all IBS subtypes are largely attributable to confounders', *Aliment Pharmacol Ther*, 40: 288-97.

Pelsers, M. M., W. T. Hermens, and J. F. Glatz. 2005. 'Fatty acid-binding proteins as plasma markers of tissue injury', *Clin Chim Acta*, 352: 15-35.

Pelsers, M. M., Z. Namiot, W. Kisielewski, A. Namiot, M. Januszkiewicz, W. T. Hermens, and J. F. Glatz. 2003. 'Intestinal-type and liver-type fatty acid-binding protein in the intestine. Tissue distribution and clinical utility', *Clin Biochem*, 36: 529-35.

Piche, T., G. Barbara, P. Aubert, S. Bruley des Varannes, R. Dainese, J. L. Nano, C. Cremon, V. Stanghellini, R. De Giorgio, J. P. Galmiche, and M. Neunlist. 2009. 'Impaired intestinal barrier integrity in the colon of patients with irritable bowel syndrome: involvement of soluble mediators', *Gut*, 58: 196-201.

Pinto-Sanchez, M. I., G. B. Hall, K. Ghajar, A. Nardelli, C. Bolino, J. T. Lau, F. P. Martin, O. Cominetti, C. Welsh, A. Rieder, J. Traynor, C. Gregory, G. De Palma, M. Pigrau, A. C. Ford, J. Macri, B. Berner, G. Bergonzelli, M. G. Surette, S. M. Collins, P. Moayyedi, and P. Bercik. 2017. 'Probiotic Bifidobacterium longum NCC3001 Reduces Depression Scores and Alters Brain Activity: A Pilot Study in Patients With Irritable Bowel Syndrome', *Gastroenterology*.

Sandler, R. S., J. E. Everhart, M. Donowitz, E. Adams, K. Cronin, C. Goodman, E. Gemmen, S. Shah, A. Avdic, and R. Rubin. 2002. 'The burden of selected digestive diseases in the United States', *Gastroenterology*, 122: 1500-11.

Schmidt, K., P. J. Cowen, C. J. Harmer, G. Tzortzis, S. Errington, and P. W. Burnet. 2015. 'Prebiotic intake reduces the waking cortisol response and alters emotional bias in healthy volunteers', *Psychopharmacology (Berl)*, 232: 1793-801.

Snaith, R. P., and A. S. Zigmond. 1986. 'The hospital anxiety and depression scale', *Br Med J (Clin Res Ed)*, 292: 344.

Suarez-Hitz, K. A., B. Otto, M. Bidlingmaier, W. Schwizer, M. Fried, and U. Ehlert. 2012. 'Altered psychobiological responsiveness in women with irritable bowel syndrome', *Psychosom Med*, 74: 221-31.

Sundin, J., I. Rangel, A. K. Kumawat, E. Hultgren-Hornquist, and R. J. Brummer. 2014. 'Aberrant mucosal lymphocyte number and subsets in the colon of post-infectious irritable bowel syndrome patients', *Scandinavian Journal of Gastroenterology*, 49: 1068-75.

Takada, M., K. Nishida, A. Kataoka-Kato, Y. Gondo, H. Ishikawa, K. Suda, M. Kawai, R. Hoshi, O. Watanabe, T. Igarashi, Y. Kuwano, K. Miyazaki, and K. Rokutan. 2016. 'Probiotic Lactobacillus casei strain Shirota relieves stress-associated symptoms by modulating the gut-brain interaction in human and animal models', *Neurogastroenterol Motil*, 28: 1027-36.

The EuroQol, Group. 1990. 'EuroQol--a new facility for the measurement of health-related quality of life. The EuroQol Group', *Health policy (Amsterdam, Netherlands)*, 16: 199-208.

van Wijck, K., T. J. Verlinden, H. M. van Eijk, J. Dekker, W. A. Buurman, C. H. Dejong, and K. Lenaerts. 2013. 'Novel multi-sugar assay for site-specific gastrointestinal permeability analysis: a randomized controlled crossover trial', *Clin Nutr*, 32: 245-51.

Vanuytsel, T., S. van Wanrooy, H. Vanheel, C. Vanormelingen, S. Verschueren, E. Houben, S. Salim Rasoel, J. Tomicronth, L. Holvoet, R. Farre, L. Van Oudenhove, G. Boeckxstaens, K. Verbeke, and J. Tack. 2014. 'Psychological stress and corticotropin-releasing hormone increase intestinal permeability in humans by a mast cell-dependent mechanism', *Gut*, 63: 1293-9.

Wells, J. M., R. J. Brummer, M. Derrien, T. T. MacDonald, F. Troost, P. D. Cani, V. Theodorou, J. Dekker, A. Meheust, W. M. de Vos, A. Mercenier, A. Nauta, and C. L. Garcia-Rodenas. 2017. 'Homeostasis of the gut barrier and potential biomarkers', *Am J Physiol Gastrointest Liver Physiol*, 312: G171-G93.

Wiklund, I. K., S. Fullerton, C. J. Hawkey, R. H. Jones, G. F. Longstreth, E. A. Mayer, R. A. Peacock, I. K. Wilson, and J. Naesdal. 2003. 'An irritable bowel syndrome-specific symptom questionnaire: development and validation', *Scandinavian Journal of Gastroenterology*, 38: 947-54.

Wong, E., G. H. Guyatt, D. J. Cook, L. E. Griffith, and E. J. Irvine. 1998. 'Development of a questionnaire to measure quality of life in patients with irritable bowel syndrome', *The European journal of surgery. Supplement.*, 583: 50-56.

Zhou, Q., W. W. Souba, C. M. Croce, and G. N. Verne. 2010. 'MicroRNA-29a regulates intestinal membrane permeability in patients with irritable bowel syndrome', *Gut*, 59: 775-84.

Zhou, Q., B. Zhang, and G. N. Verne. 2009. 'Intestinal membrane permeability and hypersensitivity in the irritable bowel syndrome', *Pain*, 146: 41-46.

Appendix I: Rome IV criteria

Diagnostic criteria for Irritable Bowel Syndrome:

Recurrent abdominal pain on average at least 1 day/week in the last 3 months, associated with two or more of the following criteria:

1. Related to defecation
2. Associated with a change in the frequency of stool
3. Associated with a change in the form (appearance) of stool

(These criteria should be fulfilled for the last 3 months with symptom onset at least 6 months prior to diagnosis.)

Diagnostic criteria for Irritable Bowel Syndrome with predominant diarrhoea:

More than one-fourth (25%) of bowel movements with Bristol stool form types 6 or 7 and less than one-fourth (25%) of bowel movements with Bristol stool form types 1 or 2.

Lacy B.E., Mearin F., Chang L., Chey W.D., Lembo A.J., Simren M., Spiller R. Bowel Disorders. Gastroenterology. 2016;150:1393–1407.

Appendix II: Questionnaire on background information

Datum för undersökningsbesök 20 ı_ı_ı. ı_ı_ı. ı_ı_ı

Bakgrundsfakta om patienten

1. Kön Man Kvinna

2. Födelsedatum 19 ı_ı_ı. ı_ı_ı. ı_ı_ı

3. Längd ı_ı_ı_ı, ı_ı cm

4. Vikt ı_ı_ı_ı, ı_ı kg

5. Vilken högsta utbildningsnivå har du?

1. Högstadium
2. År på gymnasium
3. Gymnasium
4. År på högskola
5. Examen på högskola
6. Annan utbildning, vilken? ________________

6. Har läkare konstaterat att du har någon eller några av följande sjukdomar?

Hjärtsjukdom Nej Ja

Vilken hjärtsjukdom? ______________________________

Stroke Nej Ja

Förhöjt blodtryck Nej Ja

Sockersjuka (diabetes) Nej Ja

Cancer Nej Ja

Vilken cancer? ______________________________

Smärtor i övre delen av buken (dyspepsi) Nej Ja

Laktosintolerans Nej Ja

Celiaki Nej Ja

Annan mag- eller tarmsjukdom Nej Ja

Vilken tarmsjukdom? ________________________________

Osteoporos Nej Ja

Allergi (även födoämnes-) Nej Ja

Vilken allergi? ______________________________________

Psykisk sjukdom Nej Ja

Annan långvarig sjukdom? Nej Ja

Vilken sjukdom?____________________________________

7. **Hur länge har du haft symptom på irriterad tarm?**
   1. Mindre än 6 månader
   2. 6-11 månader
   3. 1-5 år
   4. Mer än 5 år

8. **Äter du någon medicin regelbundet för behandling av irriterad tarm (t.ex. förstoppnings- och diarrémedicin, fiberpreparat)?**

   Nej Ja

   Om du har det, vilka mediciner/preparat (handelsnamn) och vilka doser (dos/frekvens) använder du?

   ____

   ____

9. **Äter du någon annan medicin regelbundet?**

   Nej Ja

   Om du har det, vilken (medicinens handelsnamn) och vilka doseringar (dos/frekvens)?

   ____

   ____

10. När använde du senast antibiotika eller annan antimikrobisk medicin som intas genom munnen (svamp-, virus-, amöbamediciner)?

Medicineringens slutdatum ı_ı_ı. ı_ı_ı. ı_ı_ı

Medicinens namn, dosering (dos/dag):

____

____

11. Har du genomgått operationer i mag- eller tarmkanalsområdet?

Nej Ja

Om det är så, vilka operationer och vilka år?

____

____

12. Har du haft inflammationer i mag-tarmkanalen (t.ex salmonella, kampylobakterier) de senastre tre åren?

Nej Ja

Om det är så, när och vad konstaterades vara orsaken till inflammationen?

____

____

Om det är så, har symptomen på irriterad tarm uppstått efter inflammationen?

Nej Ja Det kan jag inte svara på

13. Har du haft konstaterad helikobakterieinfektion?

Nej Ja

Om det är så, vilket år?

____

Om det är så, har man behandlat infektionen med antibiotika?

Nej Ja

Om man har det, har man med undersökningar försäkrat sig om att avhysningen av heliobakterien har lyckats efter avslutat vård?

Nej Ja

14. Har du någon specialdiet (t.ex. laktosfri, glutenfri eller vegetarisk?)

Nej Ja

Om du har det, vilken? ____

15. Om du har <u>konstaterad laktosintolerans</u>, äter du då låglaktos (HYLA-och INTO-produkter) eller helt laktosfri kost?

Nej Ja

16. Om du har <u>konstaterad laktosintolerans</u>, använder du laktaspreparat som kan köpas på apotek?

Regelbundet Sporadiskt Använder inte preparat

17. Äter du pre- eller probiotiska preparat?

Nej Ja

Om ja, vilka? ____

18. Röker du?

Nej Ja Sporadiskt

19. Använder du snus?

Nej Ja Sporadiskt

20. Använder du alkohol?

Dagligen Varje vecka Varje månad Mer sällan/Använder inte alkohol

<u>För Kvinnor</u>

21. Är du gravid eller ammar du?

Nej Ja

Appendix III: Food frequency questionnaire (FFQ)

(Screenshot example)

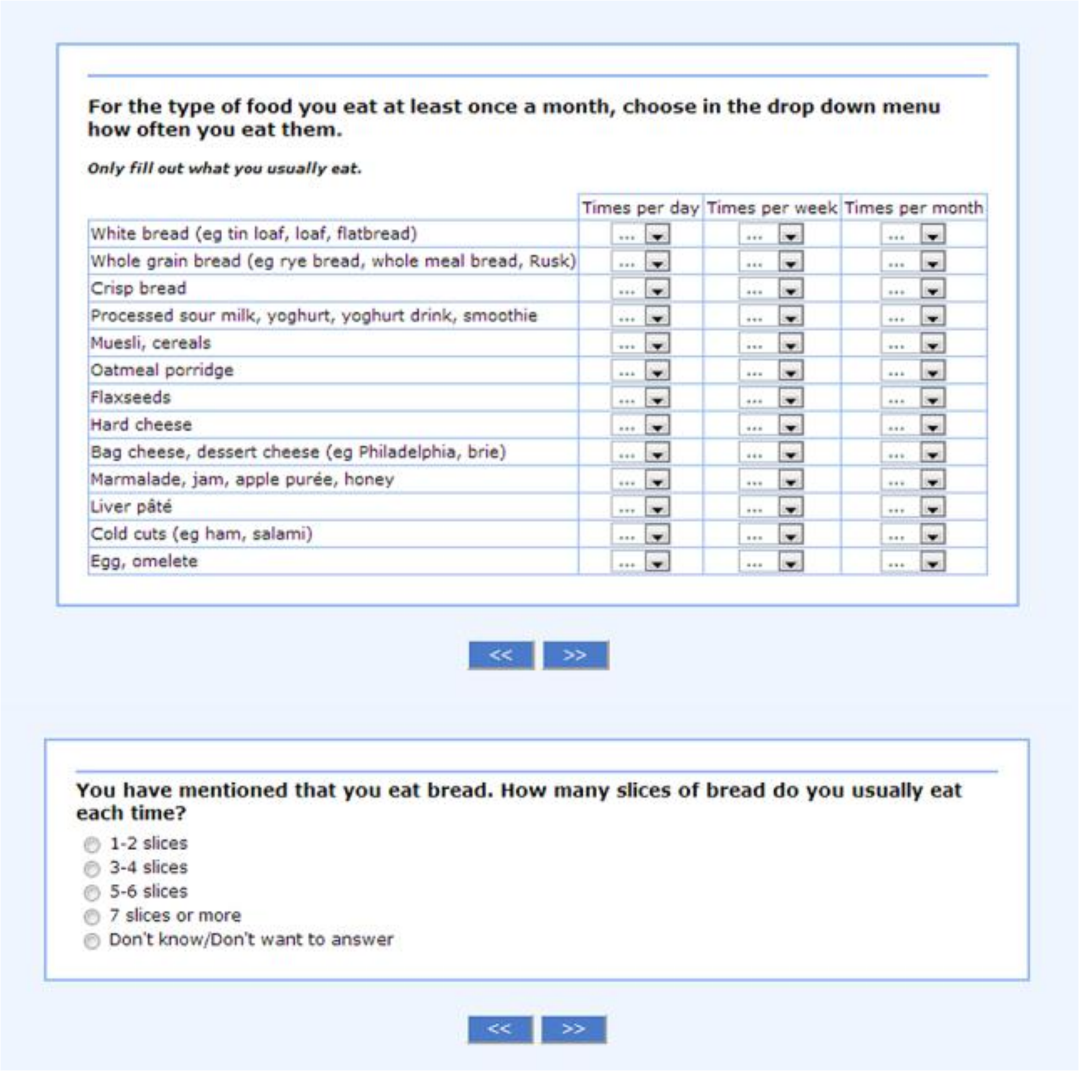

For the type of food you eat at least once a month, choose in the drop down menu how often you eat them.

Only fill out what you usually eat.

	Times per day	Times per week	Times per month
White bread (eg tin loaf, loaf, flatbread)	...	...	...
Whole grain bread (eg rye bread, whole meal bread, Rusk)	...	...	...
Crisp bread	...	...	...
Processed sour milk, yoghurt, yoghurt drink, smoothie	...	...	...
Muesli, cereals	...	...	...
Oatmeal porridge	...	...	...
Flaxseeds	...	...	...
Hard cheese	...	...	...
Bag cheese, dessert cheese (eg Philadelphia, brie)	...	...	...
Marmalade, jam, apple purée, honey	...	...	...
Liver pâté	...	...	...
Cold cuts (eg ham, salami)	...	...	...
Egg, omelete	...	...	...

<< >>

You have mentioned that you eat bread. How many slices of bread do you usually eat each time?

- 1-2 slices
- 3-4 slices
- 5-6 slices
- 7 slices or more
- Don't know/Don't want to answer

<< >>

Appendix IV: 3-day food diary

Instruktioner

Skriv ner allt du äter och dricker för tre hela dager innan ditt besök.

Ange även vad klockan är när du börjar måltiden.

Skriv sedan ett livsmedel på varje rad, och beskriv maten och livsmedlen så noggrant du kan. Beskriv exempelvis vilken sorts mjölk, fil eller yoghurt du äter eller dricker, vilket sorts ost och vilken fetthalt den har, vad bröd sorten heter och annat. Ange gärna produktnamn eller märke på produkterna.

Beskriv också hur maten är tillagad. Är maten kokt, stekt, panerad, grillad eller gratinerad?

Glöm inte tillbehör till maten, såsom senap, lingonsylt, sås, mjölk eller socker i kaffe och te eller småsaker som äts mellan måltiderna som tabletter, frukt, kakor, saft.

När du lagar maten själv kan du skriva ner de ingredienser du använt. Om du äter mat någon annan lagat kan du försöka gissa vad vilka ingredienser som används och hur maten tillagats, eller fråga den som lagat den.

Mängder kan anges i gram (om det står på en förpackning), matskedar (msk), deciliter (dl) eller stycken (st). Använd det som är enklast för dig.

Ett tips kan vara att fotografera maten för att ytterligare dokumentera!

Nedan följer ett exempel på hur intaget av mat kan dokumenteras.

Måltid	Tid	Livsmedel	Mängd (dl/g/st)
Middag	*19.00*	*Pasta*	*200 g*
		Köttfärs fetthalt 12%	*150 g*
		Tomatssås, dolmio	*2 dl*
		Permesan ost, 32 %	*10 g*
		Olivolja, ZETA	*0,25 dl*
		O,s,v….	

Studiekod: ____________________ Datum: ____________________

Dag 1-3

Måltid	Tid	Livsmedel	Mängd (dl/g/st)

Appendix V: Diary about stool frequency and consistency

Nummer: ________ Startdatum:___________________________

Instruktioner: När det kommer avföring är det meningen att du fyller i siffror och bokstaven som motsvarar vad som hände för motsvarande datum och tidpunkt.

Konsistens: Fyll i typ 1-7 (se förklaring nedan)
Känsla av ofullständig tarmtömning: Om ja, fyll i K

Exempel: Måndag kl 10: 6, K

Typ 1<br><br>Hårda klumpar, som nötter	Typ 2<br><br>Hård korv med klumpar i	Typ 3<br><br>Korv med sprickor på ytan	Typ 4<br><br>Korv, mjuk och slät	Typ 5<br><br>Mjuka bitar med tydliga kanter	Typ 6<br><br>Fluffiga bitar med otydliga kanter	Typ 7<br><br>Vattnigt, inga bitar
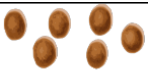	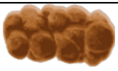	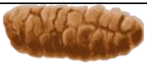	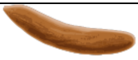	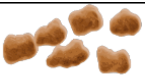	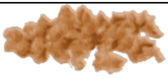	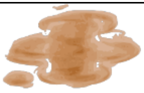

	Måndag	Tisdag	Onsdag	Torsdag	Fredag	Lördag	Söndag
**Datum ➔**							
**Tidpunkt🡻**							
**06**							
**07**							
**08**							
**09**							
**10**							
**11**							
**12**							
**13**							
**14**							
**15**							
**16**							
**17**							
**18**							
**19**							
**20**							
**21**							
**22**							
**23**							
**24**							
**01**							
**02**							
**03**							
**04**							
**05**							

Appendix VI: IBS-SSS

IBS-Symptom Severity Score

Namn ______________________________ Datum ____________ Endast för

Ålder ________ Kön (ringa in): **M** **F** Annat ________________

Summa:

Det här formuläret är utformat för att mäta och utvärdera graden av din IBS. Det är rimligt att dina symptom varierar över tid, besvara därför frågorna utifrån hur du för tillfället känner dig (dvs. under de senaste 10 dagarna ungefär). All information behandlas strikt konfidentiellt.

1. a) Lider du för tillfället av magsmärtor? Ja Nej

b) Om ja, hur kraftiga är dina magsmärtor?

0 % ——————————————— 100 %

Ingen smärta | Inte så kraftig | Ganska kraftig | Kraftig | Mycket kraftig

c) Vänligen fyll i antalet dagar som du har smärta under en 10-dagars period. Om du till exempel skriver 4 betyder det att du har smärta under 4 av 10 dagar. Om du har smärta varje dag så skriver du 10.

X 10

Antalet dagar med smärta:

2. a) Är du för tillfället uppblåst i magen? Ja Nej
(till exempel uppsvälld, svullen eller spänd i magen.
För kvinnor- ignorera eventuell uppblåsthet under menstruationen)

b) Om ja, hur allvarlig är uppblåstheten/spändheten i magen?

0 % ——————————————— 100 %

Ingen uppblåsthet | Inte så kraftig | Ganska kraftig | Kraftig | Mycket kraftig

3. Hur nöjd är du med dina avföringsmönster?

0 % ——————————————— 100 %

Mycket nöjd | Ganska nöjd | Missnöjd | Mycket missnöjd

4. Vänligen indikera på linjen nedan med ett kryss hur mycket IBS generellt sett påverkar eller stör ditt liv.

0 % ——————————————— 100 %

Inte alls | Inte mycket | Ganska mycket | Helt och hållet

Appendix VII: GSRS-IBS

THE GASTROINTESTINAL SYMPTOM RATING SCALE (GSRS) IRRITABLE BOWEL SYNDROME (IBS) VERSION

Undersökningen innehåller frågor om hur Du mår och hur Du har haft det DEN SENASTE VECKAN. Markera med ett kryss X det alternativ som bäst passar in på Dig och Din situation.

		Inga besvär alls	Obetyd-liga besvär	Milda besvär	Måttliga besvär	Ganska svåra besvär	Svåra besvär	Mycket svåra besvär
1.	Har du under den senaste veckan besvärats av ont i magen?	○	○	○	○	○	○	○
2.	Har du under den senaste veckan besvärats av smärta eller obehag i magen som blir bättre när du tömmer tarmen?	○	○	○	○	○	○	○
3.	Har du under den senaste veckan besvärats av en känsla av uppkördhet i magen?	○	○	○	○	○	○	○
4.	Har du under den senaste veckan besvärats av att du släpper ut gaser?	○	○	○	○	○	○	○
5.	Har du under den senaste veckan besvärats av förstoppning? (problem med att tömma tarmen.)	○	○	○	○	○	○	○
6.	Har du under den senaste veckan besvärats av många tarmtömningar per dag?	○	○	○	○	○	○	○
7.	Har du under den senaste veckan besvärats av lös avföring?	○	○	○	○	○	○	○
8.	Har du under den senaste veckan besvärats av hård avföring?	○	○	○	○	○	○	○
9.	Har du under den senaste veckan besvärats av trängande avföringsbehov? (bråttom till toaletten.)	○	○	○	○	○	○	○
10.	Har du under den senaste veckan besvärats av en känsla av att du inte riktigt kan tömma tarmen?	○	○	○	○	○	○	○
11.	Har du under den senaste veckan besvärats av att du känt dig mätt innan du har ätit färdigt? (blir fort mätt.)	○	○	○	○	○	○	○
12.	Har du under den senaste veckan besvärats av att du känt dig mätt länge efter att du ätit (färdigt)?	○	○	○	○	○	○	○
13.	Har du under den senaste veckan besvärats av att magen svullnar så att det syns?	○	○	○	○	○	○	○

Appendix VIII: IBS-QOL

Om hur du känner dig

Tänk på ditt liv under den **senaste månaden (senaste 30 dagarna)**, och titta på påståendena nedan. Varje påstående har fem olika svar. För varje påstående, ringa in det svar som bäst beskriver dina känslor.

	Inte alls 1	Lite 2	Måttligt 3	Mycket 4	Väldigt mycket 5
1. Jag känner mig hjälplös på grund av mina tarmproblem.	○	○	○	○	○
2. Jag är generad över lukten orsakad av mina tarmproblem.	○	○	○	○	○
3. Jag är besvärad av hur mycket tid som jag spenderar på toaletten.	○	○	○	○	○
4. Jag känner mig sårbar för andra sjukdomar på grund av mina tarmproblem.	○	○	○	○	○
5. Jag känner mig tjock/uppsvälld på grund av mina tarmproblem.	○	○	○	○	○
6. Jag känner att jag håller på att tappa kontrollen över mitt liv på grund av mina tarmproblem.	○	○	○	○	○
7. Jag känner att mitt liv känns mindre njutbart på grund av mina tarmproblem.	○	○	○	○	○
8. Jag känner mig obehaglig till mods när jag talar om mina tarmproblem.	○	○	○	○	○
9. Jag känner mig nedstämd på grund av mina tarmproblem.	○	○	○	○	○
10. Jag känner mig isolerad från andra på grund av mina tarmproblem.	○	○	○	○	○
11. Jag måste se upp med den mängd mat jag äter på grund av mina tarmproblem.	○	○	○	○	○
12. Sexuella aktiviteter/intimitet är besvärligt för mig på grund av mina tarmproblem. *(Om ej tillämplig, markera in "INTE ALLS").*	○	○	○	○	○
13. Jag känner mig arg för att jag har tarmproblem.	○	○	○	○	○
14. Jag känner att jag irriterar andra på grund av mina tarmproblem.	○	○	○	○	○
15. Jag oroar mig för att mina tarmproblem skall förvärras.	○	○	○	○	○
16. Jag känner mig lättretlig på grund av mina tarmproblem.	○	○	○	○	○

	Inte alls	Lite	Måttligt	Mycket	Väldigt mycket
	1	2	3	4	5
17. Jag oroar mig för att folk tycker att jag överdriver mina tarmproblem.	○	○	○	○	○
18. Jag känner att jag får mindre gjort på grund av mina tarmproblem.	○	○	○	○	○
19. Jag måste undvika stressande situationer på grund av mina tarmproblem.	○	○	○	○	○
20. Mina tarmproblem minskar sexlusten.	○	○	○	○	○
21. Mina tarmproblem begränsar vad jag kan ha på mig.	○	○	○	○	○
22. Jag måste undvika fysiskt ansträngande aktiviteter på grund av mina tarmproblem.	○	○	○	○	○
23. Jag måste se upp med vilken sorts mat som jag äter på grund av mina tarmproblem.	○	○	○	○	○
24. På grund av mina tarmproblem har jag svårt för att vara bland folk jag inte känner väl.	○	○	○	○	○
25. Jag känner mig slö på grund av mina tarmproblem.	○	○	○	○	○
26. Jag känner mig inte riktigt fräsch på grund av mina tarmproblem.	○	○	○	○	○
27. Långa resor är svåra för mig på grund av mina tarmproblem.	○	○	○	○	○
28. Jag känner mig frustrerad över att jag inte kan äta när jag vill på grund av mina tarmproblem.	○	○	○	○	○
29. Det är viktigt att vara nära en toalett på grund av mina tarmproblem.	○	○	○	○	○
30. Mitt liv kretsar kring mina tarmproblem.	○	○	○	○	○
31. Jag oroar mig över att förlora kontrollen över mina tarmtömningar.	○	○	○	○	○
32. Jag är rädd för att jag inte skall kunna ha en tarmtömning.	○	○	○	○	○
33. Mina tarmproblem påverkar mina närmaste relationer.	○	○	○	○	○
34. Jag tycker att ingen förstår mina tarmproblem.	○	○	○	○	○

Appendix IX: 5Q-5D-5L

Hälsoenkät

Svensk version för Sverige

(Swedish version for Sweden)

Kryssa under varje rubrik bara i EN ruta som bäst beskriver din hälsa IDAG.

RÖRLIGHET

Jag har inga svårigheter med att gå omkring ❑
Jag har lite svårigheter med att gå omkring ❑
Jag har måttliga svårigheter med att gå omkring ❑
Jag har stora svårigheter med att gå omkring ❑
Jag kan inte gå omkring ❑

PERSONLIG VÅRD

Jag har inga svårigheter med att tvätta mig eller klä mig ❑
Jag har lite svårigheter med att tvätta mig eller klä mig ❑
Jag har måttliga svårigheter med att tvätta mig eller klä mig ❑
Jag har stora svårigheter med att tvätta mig eller klä mig ❑
Jag kan inte tvätta mig eller klä mig ❑

VANLIGA AKTIVITETER *(t ex arbete, studier, hushållssysslor, familje- eller fritidsaktiviteter)*

Jag har inga svårigheter med att utföra mina vanliga aktiviteter ❑
Jag har lite svårigheter med att utföra mina vanliga aktiviteter ❑
Jag har måttliga svårigheter med att utföra mina vanliga aktiviteter ❑
Jag har stora svårigheter med att utföra mina vanliga aktiviteter ❑
Jag kan inte utföra mina vanliga aktiviteter ❑

SMÄRTOR/BESVÄR

Jag har varken smärtor eller besvär ❑
Jag har lätta smärtor eller besvär ❑
Jag har måttliga smärtor eller besvär ❑
Jag har svåra smärtor eller besvär ❑
Jag har extrema smärtor eller besvär ❑

ORO/NEDSTÄMDHET

Jag är varken orolig eller nedstämd ❑
Jag är lite orolig eller nedstämd ❑
Jag är ganska orolig eller nedstämd ❑
Jag är mycket orolig eller nedstämd ❑
Jag är extremt orolig eller nedstämd ❑

Bästa hälsa du kan tänka dig

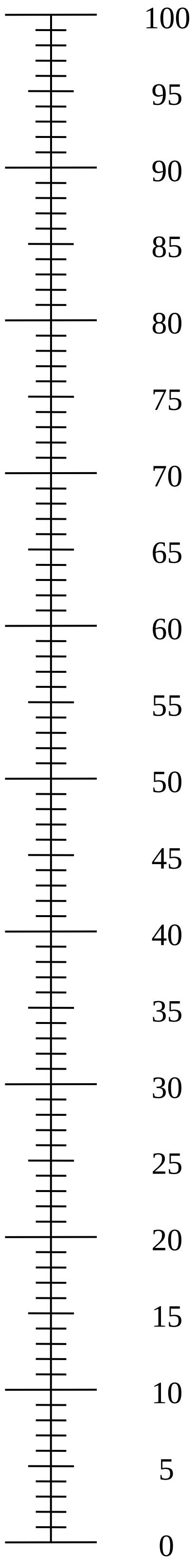

Sämsta hälsa du kan tänka dig

- Vi vill veta hur bra eller dålig din hälsa är IDAG.
- Den här skalan är numrerad från 0 till 100.
- 100 är den bästa hälsa du kan tänka dig.
  0 är den sämsta hälsa du kan tänka dig.
- Sätt ett X på skalan för att visa hur din hälsa är IDAG.

Skriv nu i rutan nedan det nummer du har markerat på skalan.

Appendix X: HADS

Fyll i det alternativ som stämmer bäst in på Dig!

Jag känner mig spänd eller nervös:
( ) Mestadels
( ) Ofta
( ) Av och till
( ) Inte alls

Jag uppskattar fortfarande saker jag tidigare uppskattat:
( ) Definitivt lika mycket
( ) Inte lika mycket
( ) Endast delvis
( ) Nästan inte alls

Jag har en känsla av att något hemskt kommer att hända:
( ) Mycket klart och obehagligt
( ) Inte så starkt nu
( ) Betydligt svagare nu
( ) Inte alls

Jag kan skratta och se det roliga i saker och ting:
( ) Lika ofta som tidigare
( ) Inte lika ofta nu
( ) Betydligt mer sällan nu
( ) Aldrig

Jag bekymrar mig över saker:
( ) Mestadels
( ) Ganska ofta
( ) Av och till
( ) Någon enstaka gång

Jag känner mig på gott humör:
( ) Aldrig
( ) Sällan
( ) Ibland
( ) Mestadels

Jag kan sitta stilla och känna mig avslappnad:
( ) Definitivt
( ) Vanligtvis
( ) Sällan
( ) Aldrig

Allting känns trögt:
( ) Nästan alltid
( ) Ofta
( ) Ibland
( ) Aldrig

Jag känner mig orolig, som om jag har ”fjärilar” i magen:
( ) Aldrig
( ) Ibland
( ) Ganska ofta
( ) Väldigt ofta

Jag har tappat intresset för hur jag ser ut:
( ) Fullständigt
( ) Till stor del
( ) Delvis
( ) Inte alls

Jag känner mig rastlös:
( ) Väldigt ofta
( ) Ganska ofta
( ) Sällan
( ) Inte alls

Jag ser med glädje fram emot saker och ting:
( ) Lika mycket som tidigare
( ) Mindre än tidigare
( ) Mycket mindre än tidigare
( ) Knappast alls

Jag får plötsliga panikkänslor:
( ) Väldigt ofta
( ) Ganska ofta
( ) Sällan
( ) Aldrig

Jag kan uppskatta en god bok, eller ett TV- eller radioprogram
( ) Ofta
( ) Ibland
( ) Sällan
( ) Mycket sällan

Appendix XI: Perceived Stress Scale (PSS)

Frågorna i den här skalan handlar om dina känslor och tankar **under den senaste månaden**. För varje fråga ombeds du markera med en cirkel hur ofta du kännt eller tänkt på ett visst sätt.

Namn _______________________________________ Datum ___________

Ålder _______ Kön (ringa in): **M** **F** Annat _____________________

0 = Aldrig **1 = Nästan aldrig** **2 = Ibland** **3 = Ganska ofta** **4 = Mycket ofta**

1. Under den senaste månaden, hur ofta har du varit upprörd
på grund av en oförutsedd händelse? 0 1 2 3 4

2. Under den senaste månaden, hur ofta har du känt att du var
oförmögen att hantera viktiga saker i ditt liv? 0 1 2 3 4

3. Under den senaste månaden, hur ofta har du känt dig nervös
eller ”stressad”? 0 1 2 3 4

4. Under den senaste månaden, hur ofta har du känt dig säker
på din förmåga att hantera personliga problem? 0 1 2 3 4

5. Under den senaste månaden, hur ofta har du känt att saker
har blivit som du vill? 0 1 2 3 4

6. Under den senaste månaden, hur ofta har du upptäckt att du
inte kunat hantera alla saker du måste göra? 0 1 2 3 4

7. Under den senaste månaden, hur ofta har du kunnat hantera
irritationer i ditt liv? 0 1 2 3 4

8. Under den senaste månaden, hur ofta har du känt att du har
läget under kontroll? 0 1 2 3 4

9. Under den senaste månaden, hur ofta har du blivit upprörd
över saker som legat utanför vad du kan påverka? 0 1 2 3 4

10. Under den senaste månaden, hur ofta har du känt att
svårigheter blivit så stora att du inte kunnat lösa dem? 0 1 2 3 4

Mind Garden, Inc.
info@mindgarden.com
www.mindgarden.com
